# Allosteric modulation of protein kinase A in individuals affected by NLPD‐PKA, a neurodegenerative disease in which the PRKAR1B L50R variant is expressed

**DOI:** 10.1111/febs.70098

**Published:** 2025-04-17

**Authors:** Tal Benjamin‐Zukerman, Valeria Pane, Rania Safadi‐Safa, Meir Solomon, Varda Lev‐Ram, Mohammad Aboraya, Anwar Dakwar, Daniela Bertinetti, Andrew Hoy, Merel O. Mol, John van Swieten, Rodrigo Maillard, Friedrich W. Herberg, Ronit Ilouz

**Affiliations:** ^1^ The Azrieli Faculty of Medicine Bar Ilan University Safed Israel; ^2^ Department of Biochemistry University of Kassel Germany; ^3^ Department of Pharmacology University of California, San Diego La Jolla CA USA; ^4^ Department of Chemistry Georgetown University Washington DC USA; ^5^ Department of Neurology and Alzheimer Center Erasmus MC Erasmus University Medical Center Rotterdam The Netherlands; ^6^ The Leslie & Susan Goldschmied (Gonda) Multidisciplinary Brain Research Center Bar‐Ilan University Ramat‐Gan Israel

**Keywords:** allostery, holoenzyme assembly, neurodegenerative disease, *PRKAR1B*, protein kinase A

## Abstract

Protein kinase A (PKA) is a crucial signaling enzyme in neurons, with its dysregulation being implicated in neurodegenerative diseases. Assembly of the PKA holoenzyme, comprising a dimer of heterodimers of regulatory (R) and catalytic (C) subunits, ensures allosteric regulation and functional specificity. Recently, we defined the RIβ‐L50R variant as a causative mutation that triggers protein aggregation in a rare neurodegenerative disease, neuronal loss, and parkinsonism driven by a PKA mutation (NLPD‐PKA). However, the mechanism underlying uncontrolled PKA allosteric regulation and its connection to the functional outcomes leading to clinical symptoms remains elusive. In this study, we established an *in vitro* model using patient‐derived cells for a personalized approach and employed direct measurements of purified proteins to investigate disease mechanisms in a controlled environment. Structural analysis and circular dichroism spectroscopy revealed that cellular protein aggregation resulted from misfolded RIβ‐subunits, preventing holoenzyme assembly and anchoring through A‐kinase anchoring proteins (AKAPs). While maintaining high affinity to the C‐subunit, the resulting RIβ‐L50R:C heterodimer exhibits reduced cooperativity, requiring lower cAMP concentrations for dissociation. Consequently, there was an increased translocation of the C‐subunit into the nucleus, impacting gene expression. We successfully controlled C‐subunit translocation by introducing a mutation that decreased RIβ:C dissociation in response to elevated cAMP levels. This research thus sets the stage for developing therapeutic strategies that modulate PKA assembly and allostery, thus exerting control over the unique molecular signatures identified in the disease‐associated transcriptome profile.

## Graphical abstract

In healthy neurons, Protein kinase A (PKA) forms a tetrameric holoenzyme enabling precise allosteric regulation. We characterized a novel neurodegenerative disease, neuronal loss, and parkinsonism driven by PRKAR1B mutation (NLPD‐PKA), demonstrating disrupted holoenzyme assembly, reduced cooperativity, and increased catalytic subunit nuclear translocation, altering gene expression in patient‐derived cells. Our findings set the stage for therapeutic strategies targeting PKA assembly to control disease‐specific transcriptomic signatures.

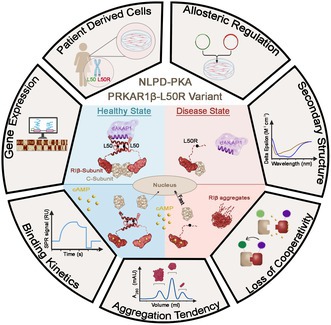

**Abbreviations**
AKAPs: A‐kinase anchoring proteins
BRET2: bioluminescence resonance energy transfer 2
cAMP: cyclic adenosine monophosphate
CD: circular dichroism
CNB: cyclic nucleotide binding
C‐subunit: catalytic subunit
D/D: dimerization and docking
FP: fluorescence polarization
FSK: forskolin
IBMX: isobutylmethylxanthine
IF: immunofluorescence
IHC: immunohistochemistry
ISO: isoproterenol
MRI: magnetic resonance imaging
NLPD‐PKA: neuronal loss and parkinsonism driven by a PKA mutation
PKA: protein kinase A
PRKAR1B: protein kinase cAMP‐dependent type I regulatory subunit beta
RNA‐seq: RNA sequencing
SEC: size‐exclusion chromatography
SPR: surface plasmon resonance
WB: western blot
WT: wild type

## Introduction

Protein Kinase A (PKA) is an important signaling enzyme that plays a fundamental role in diverse neuronal functions including synaptic plasticity, neuronal development, dopamine synthesis, and long‐term memory [1, 2, 3, 4, 5]. PKA dysfunction contributes to the cognitive decline observed in many neurodegenerative diseases such as Alzheimer's disease (AD), Parkinson's disease (PD), and amyotrophic lateral sclerosis (ALS) [6, 7, 8]. In AD, PKA is implicated in neurofibrillary pathology, where increased tau phosphorylation by PKA leads to tau aggregation and toxicity [9, 10]. Modulation of Leucine‐rich repeat kinase 2 (LRRK2) activity through PKA phosphorylation is linked to the progression of PD [11, 12], while reduced PKA activity is seen in the spinal cords of patients with ALS due to loss of PKA‐rich motor neurons [13]. Moreover, abnormal PKA pathway regulation is also observed in Huntington disease progression [14].

PKA function is regulated by the assembly of isoform‐specific holoenzymes localized to specific cellular sites [15]. In the inactive state, PKA exists as a holoenzyme that comprises a regulatory (R) subunit dimer and two catalytic (C) subunits which together are assembled into a quaternary structure (R_2_:C_2_) [16]. In humans, four catalytic isoforms (Cα, Cβ, Cγ, and PrKX) are known, as are four regulatory subunits (RIα, RIβ, RIIα, and RIIβ). The crystal structures of RIα_2_:C_2_, RIβ_2_:C_2_, and RIIβ_2_:C_2_ revealed how isoform‐specific assembly can create distinct quaternary structures that allow functional non‐redundancy of the regulatory isoforms [16, 17, 18]. The various PKA holoenzymes differ in terms of their allosteric mechanism and sensitivity to cAMP, as well as their putative associations with diseases resulting from the appearance of the R‐subunit variants. While the incorporation of different R‐subunits is linked to various maladies, only mutations in the *PRKAR1B* gene, encoding for the RIβ‐subunit, are linked with neurodevelopmental or neurodegenerative diseases [19, 20]. Functional specificity of RIβ is provided by the precise localization of this subunit within various brain regions [17]. PKA holoenzymes are typically targeted at specific microdomains within the cell via interaction of the R‐subunit with A‐Kinase Anchoring Proteins (AKAPs) [21].

All R‐subunits share similar multi‐domain organization. Each R‐subunit consists of a Dimerization and Docking (D/D) domain in the N‐terminal region, followed by an inhibitor sequence, and two tandem cyclic nucleotide‐binding domains (referred to as CNB‐A and CNB‐B) that bind cAMP and activate the enzyme. The D/D domain forms an isologous dimeric structure crucial for assembling the PKA holoenzyme into a dimer comprising two R:C heterodimers. Additionally, the D/D domain functions as a docking site for AKAPs. Given the central role of the D/D domain in maintaining the structural integrity of the PKA holoenzyme, this region is evolutionarily conserved [22, 23]. In contrast to the abundance of known variants affecting the CNB domains, only a few variants affecting this region have been observed. Strikingly, such variants are associated with pathogenic effects [20, 24].

Recently, we defined the RIβ‐L50R variant as a causative agent driving an age‐dependent behavioral and neurodegenerative disease phenotype in human and mouse models. We proposed the name Neuronal Loss and Parkinsonism Driven by a PKA mutation (NLPD‐PKA) for this condition, given how it encapsulates the key pathological features observed in individuals with this disease. Mechanistically, this mutation disrupts RIβ dimerization, leading to RIβ aggregation of its monomers in both human and mouse brains [25].

In the present study, we investigated several pathogenic RIβ variants presenting mutations within the D/D domain, defining RIβ‐L50R as an exclusive variant able to disrupt RIβ dimerization and the assembly of PKA holoenzyme as demonstrated with cells derived from patients expressing the heterozygous L50R mutant. Furthermore, we observed abnormal cooperativity in PKA holoenzyme activation using recombinant proteins. Comprehensive analysis relying on structural, biophysical, biochemical, and cellular approaches revealed how the L50R‐encoding variation destabilizes the D/D domain, resulting in protein misfolding and increased oligomerization. Specifically, we found that RIβ‐L50R forms a heterodimer (RIβ:C), rather than a dimer of heterodimers (RIβ_2_:C_2_), with increased sensitivity to cAMP. Such heterodimer dynamics dysregulation was reflected in altered RNA expression patterns, thus providing a signature of neurodegenerative disease driven by the RIβ‐L50R variant.

## Results

### Structural analysis of RIβ‐subunit variants

**Fig. 1:**
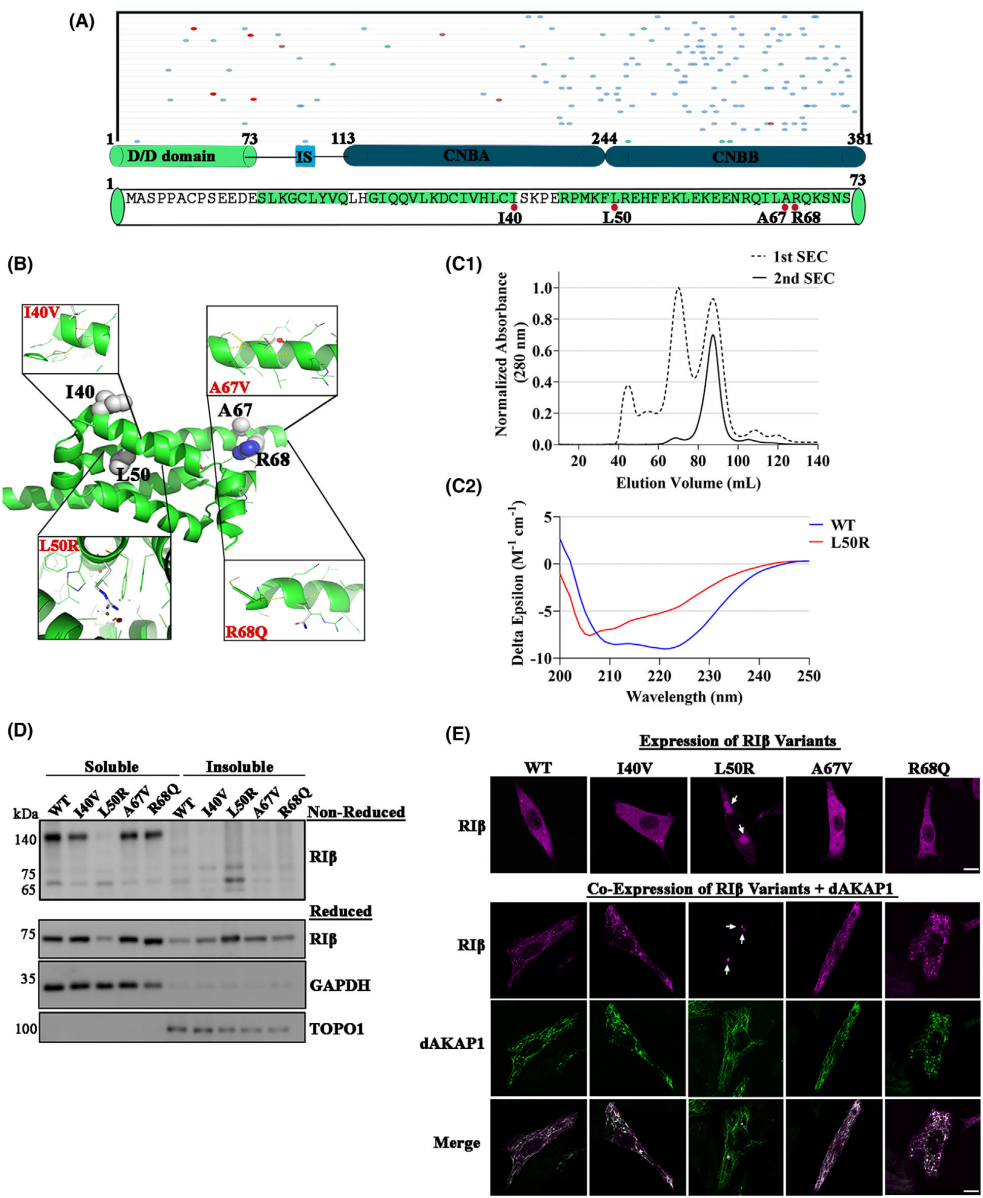
*PRKAR1B* variants. (A) *PRKAR1B* domain structure encompasses a dimerization and docking (D/D) domain, succeeded by an inhibitor sequence and a pair of cAMP‐binding domains. Displayed below are all the identified variants of human *PRKAR1B*, sourced from the UniProt database. Variants that have been identified as likely pathogenic or confirmed pathogenic are marked with circles. Red circles pinpoint the variants that are the focus of this manuscript. The sequence for the D/D domain is presented, with the residues being investigated in this work accentuated. Helices A0, A1, and A3 from each protomer contribute to dimerization. (B) In each R‐subunit, the D/D domain establishes an isologous dimer. This arrangement features identical binding sites on both subunits that interface complementarily when rotated by 180° in relation to one another. Disease‐related residues are represented as spheres. Detailed views of individual residues under investigation, visualized using PyMOL, are shown in boxes. (C1) After its purification, the 1st size‐exclusion chromatography (SEC) elution profile (*n* = 2) for the D/D domain displays multiple peaks likely corresponding to multimeric species. The dimeric D/D is expected to elute at ~ 90 mL. Collection of ~ 90 mL peak and re‐injection to SEC (*n* = 2) does not show repopulate of multimeric species, indicating that the dimeric D/D domain is stable. (C2) Circular dichroism (CD) spectrum of the wildtype D/D domain (*n* = 13) displays two minima at 208 and 222 nm, which is characteristic of α‐helical proteins. The CD spectrum of L50R (*n* = 9) loses the typical features of α‐helices, indicating that this mutation disrupts the D/D domain fold. Deconvolution of the CD data indicates that the wildtype and L50R proteins have 87% and 43% helicity, respectively. (D) PC12 cells were co‐transfected to express mKO2‐tagged RIβ‐WT or RIβ variants and mCerulean‐dAKAP1. Cell lysates from three independent experiments divided into soluble and insoluble fractions which were separated by SDS/PAGE under both non‐reduced (upper gel) or reduced conditions (lower gel). Western blot analysis was performed with GAPDH serving as a loading control for the soluble fraction and TOPO1 as a loading control for the insoluble fraction. (E) Confocal microscopy images depict transiently transfected PC12 cells expressing mKO2‐tagged RIβ‐WT or mutants (upper panel) and the same cells were co‐transfected by using mKO2‐tagged RIβ‐WT or mutants and dAKAP1(lower panel). Representative Images from three independent experiments were captured at 63× magnification, with arrows highlighting the aggregates. The scale bar represents 10 μm. CNB; cyclic nucleotide‐binding domain.

The RIβ‐subunit comprises a dimerization and docking (D/D) domain, followed by an inhibitor sequence and two cAMP‐binding domains. Figure 1A highlights all identified human RIβ variants found at the UniProt database. Variants that have been identified as likely pathogenic or confirmed as pathogenic are denoted. In our analysis, we identified a pattern to the RIβ variants, where changes are primarily clustered within the cAMP‐binding domains. In contrast, the D/D domain contained fewer such differences. The limited distribution of modified residues suggests that conservation of the RIβ sequence is fundamental for maintaining the structural integrity and functional stability of the holoenzyme. To understand the pathological consequences of genetic diversity within the *PRKAR1B*‐encoding RIβ D/D domain and its influence on PKA regulatory mechanisms, we focused our analysis on four specific variants, namely, the I40V, L50R, A67V, and R68Q (positions indicated with red circles in Fig. 1A).

We conducted structural analysis and *in‐silico* modeling to investigate these four amino acid substitutions. Employing the PyMOL Mutagenesis Wizard, we implemented these replacements on the solved D/D domain structure (structure PDB ID: 4F9K). Dimerization of RIβ occurs when two monomers interlock to form a symmetrical structure consisting of three α‐helices (structure PDB ID: 4F9K). Within each monomer, hydrophobic and polar residues cooperate to maintain dimerization. The hydrophobic groove formed by these two protomers serves as a docking site for AKAPs. We hypothesized that RIβ variants I40V, A67V, and R68Q, in which the modified residues are exposed to the surrounding solution, might not be affected in terms of dimer formation nor AKAP binding (Fig. 1B). However, during the *in‐silico* modeling of RIβ variants, where we made efforts to minimize clashes between residues, it became clear that in the L50R replacement could not avoid clashes with the surrounding residues, suggesting that this mutation may disrupt dimer formation (Fig. 1B). This finding emphasizes the potential significance of the evolutionary conserved L50 in preserving dimerization and hence, the integrity of holoenzyme assembly, subsequently allowing for allosteric regulation. To validate the *in‐silico* modeling, we used CD spectroscopy to study the secondary structure of the native RIβ and L50R variant D/D domains. Purified dimeric native RIβ D/D domain (Fig. 1C) has a typical CD spectrum for an α‐helical protein, with two minima at 208 and 222 nm. However, L50R displayed loss of absorption at both 208 and 222 nm, indicating a dramatic loss of α‐helices. In fact, a quantitative analysis of the CD spectra revealed that RIβ wildtype (RIβ‐WT) has 87% of α‐helices (in agreement with the high‐resolution structure PDB ID: 4F9K) whereas the mutant has 43%. Thus, the loss of α‐helical content for L50R provides evidence by which the full‐length protein is unable to form dimers in solution like RIβ‐WT. We found that the mutation disrupts the secondary structure of the three α‐helices that form the D/D domain fold. The significant loss of α‐helical content in the monomeric L50R mutant indicates that dimerization and folding might be coupled.

### Impact of *PRKAR1B* variants on R‐subunit homodimerization in the cell

To validate the structural results, we performed biochemical and cellular analyses on mutant RIβ proteins. Accordingly, we introduced DNA encoding the I40V, L50R, A67V, or R68Q variants into a mKO2 fluorescently tag‐encoding vector and transiently overexpressed the RIβ‐WT or mutant RIβ in dissociated PC12 cells. After resolving the soluble and insoluble protein fractions from transfected cell lysates and separating their contents by SDS/PAGE under non‐reducing conditions, RIβ‐WT and the I40V, A67V, R68Q mutants were detected as dimers in the soluble fractions. The L50R variant, however, was found in the insoluble fraction as monomers (Fig. 1D, gel denoted as non‐reduced). When the same proteins were separated by SDS/PAGE under reducing conditions, it was noted that only the L50R replacement caused RIβ to aggregate as it was found in the insoluble fraction. The other RIβ mutant proteins were mostly soluble (Fig. 1D, gel denoted as reduced). In these experiments, GAPDH and TOPO1 were used as markers to ensure the efficiency of separation of the soluble and insoluble fractions, respectively.

### Impact of RIβ variants on RIβ‐dAKAP1 interaction and subcellular localization

To further investigate the RIβ variants and their subcellular localization when expressed alone or in the presence of dAKAP1, we co‐transfected PC12 cells to individually express mKO2‐tagged RIβ‐WT or the I40V, L50R, A67V, or R68Q RIβ variants (Fig. 1E, upper images) or together with mCerulean‐tagged dAKAP1 (Fig. 1E, lower images; red for RIβ variants, green for dAKAP1). Consistent with the biochemical results presented in Fig. 1D, the RIβ protein variants in the soluble fraction exhibited a diffused cytoplasmic pattern in the immunofluorescence (IF) images. Conversely, the RIβ‐L50R variant detected in the insoluble fraction was observed in aggregates in the IF images (Fig. 1E, upper images). Due to its impaired dimerization, we hypothesized that the L50R variant disrupted the co‐localization of the RIβ proteins with dAKAP1 protein in the mitochondria, given how the dAKAP1‐binding site is found in the dimerization interface. On the other hand, although the modified I40V, A67V, R68Q and residues are located at the hydrophobic groove, our *in‐silico* modeling did not predict any interference with AKAP binding, despite their proximity to the crucial binding site. As anticipated, the co‐expression of the native protein or the I40V, A67V, R68Q RIβ protein variants with dAKAP1 led to the recruitment of RIβ to the mitochondria (Fig. 1E). However, in accordance with our prediction, RIβ‐L50R did not co‐localize with dAKAP1 at the mitochondria. These results emphasize the importance of the L50 residue in influencing the interaction between RIβ and dAKAP1, revealing the molecular intricacies of this association.

### Patient‐derived cells as an *in vitro* model for the neurodegenerative disease NLPD‐PKA

**Fig. 2:**
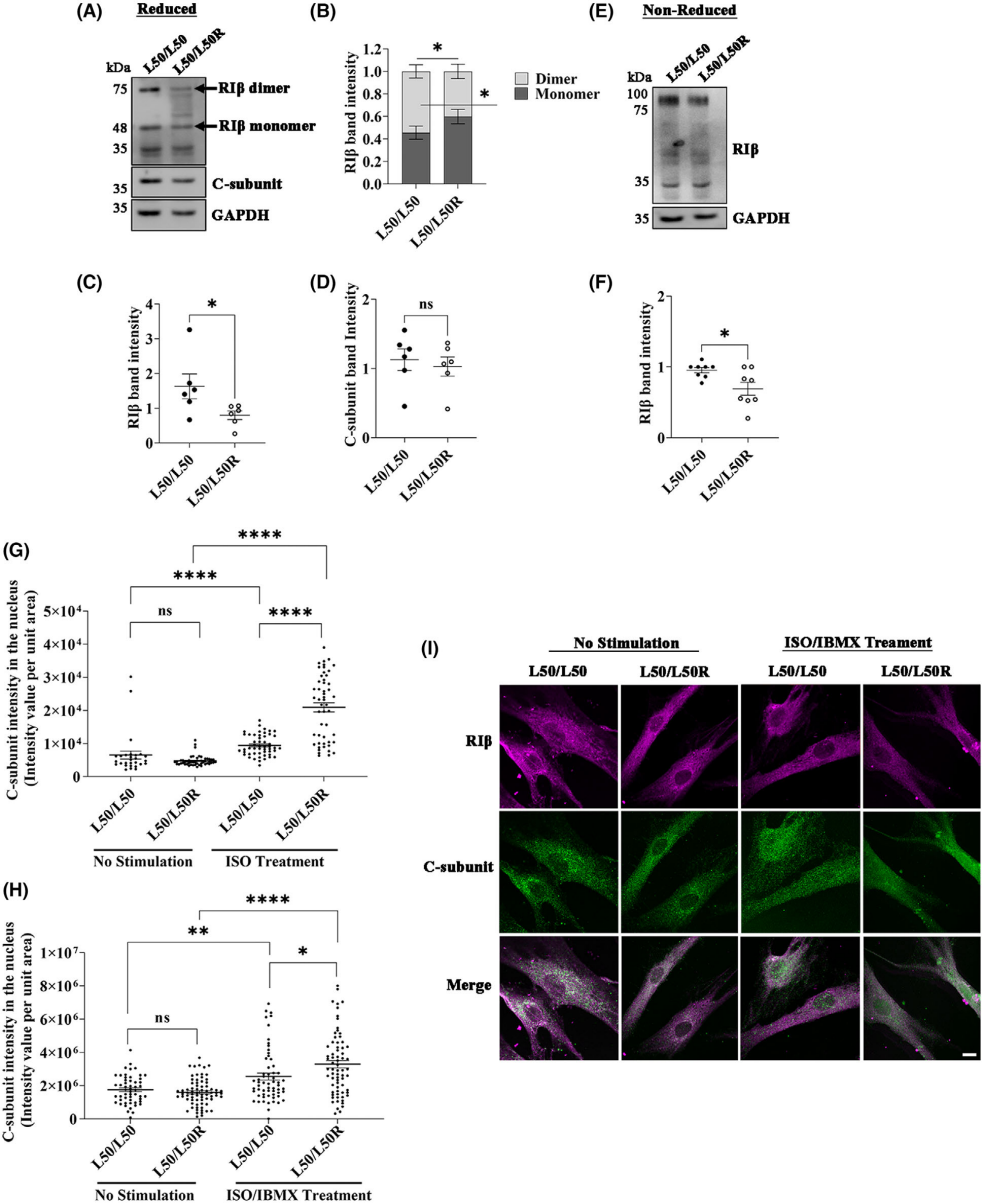
Impact of the L50/L50R heterozygous mutation on RIβ dimerization and C‐subunit nuclear translocation in patient‐derived cells. (A) Comparison of RIβ protein expression in cell lysates from a healthy individual (L50/L50) and a patient with the L50R heterozygous mutation (L50/L50R). Lysates from six independent experiments were subjected to SDS/PAGE under reducing conditions. Arrows indicate the migration of RIβ proteins in both monomer and dimer forms. The C‐subunit expression was determined using a specific antibody. GAPDH was used as a loading control. (B) Quantification of RIβ monomer‐to‐dimer ratios from six independent experiments. RIβ band intensities were normalized to GAPDH for each experiment. Data are presented as the mean ± standard error (SE), with statistical significance assessed using the Mann–Whitney test (*P ≤ 0.05). (C) Quantification of total RIβ protein expression, normalized to GAPDH, based on band intensities from six independent experiments. Data are shown as the mean ± SEM, with statistical analysis performed using the Mann–Whitney test (*P ≤ 0.05). (D) Quantification of total C‐subunit protein expression, normalized to GAPDH, based on band intensities from six independent experiments, as performed in panel C. (E) A representative SDS/PAGE gel from six independent experiments under non‐reducing conditions was used to compare the total expression levels of the dimeric form of RIβ between L50/L50 and L50/L50R patients. (F) Total expression of RIβ protein expression from the gel run in E was quantified based on band intensity from 6 independent experiments. Mann–Whitney test was performed *P ≤ 0.05. Data are presented as the mean ± standard error of the mean (SEM). (G) Quantification of the C‐subunit intensity in the nucleus before and after 30 min of Isoproterenol (ISO) treatment. Each dot represents an individual cell. Data are presented as the mean ± standard error of the mean (SEM), with statistical significance determined using an unpaired *t*‐test (****P ≤ 0.0001; ns, not significant). Three independent experiments. (H) As in G, but cells were treated with a combination of ISO and Isobutylmethylxanthine (IBMX) for 30 min. Data are presented as the mean ± standard error of the mean (SEM), with statistical significance determined using an unpaired *t*‐test (*P ≤ 0.05, **P ≤ 0.01, ****P ≤ 0.0001). (I) Representative images from the experiment in H showing patient‐derived cells before and after treatment with 1 μm ISO combined with 200 μm IBMX. DMSO was used as the control treatment. Images were captured 30 min post‐treatment at 63× magnification. Scale bar: 10 μm.

To investigate the molecular and cellular mechanisms of the disease associated with the RIβ‐L50R variant, i.e., NLPD‐PKA, we performed analyses using primary fibroblasts derived from skin biopsies of both a healthy individual (L50/L50) and a patient heterozygous for the mutation (L50/L50R). These cells capture the chronological and biological aging aspects of the patients. The cells were obtained from a female patient aged 54 years for whom we have previously presented MRI scans and clinical symptoms associated with this individual, along with an age‐ and sex‐matched healthy individual [25]. We initially performed biochemical analyses on cell lysates extracted from these primary cells. The lysates were loaded and separated by SDS/PAGE under reduced conditions to determine the ratio between monomeric and dimeric forms of RIβ, as well as to evaluate the total expression levels of RIβ‐ and C‐subunits. In the patient expressing the RIβ‐L50R variant, expression levels of RIβ proteins in the lysates were lower than in those from the healthy control. Furthermore, there was reduced presence of RIβ proteins in their dimeric form, as compared to the healthy individual (Fig. 2A–C). The distribution between monomers and dimers was quantified based on RIβ band intensities (Fig. 2B). Importantly, the *PRKAR1B* mutation leading to the appearance of the RIβ‐L50R variant did not impact C‐subunit expression levels (Fig. 2D). GAPDH was used to ensure equal protein loading on the gels (Fig. 2A). Considering that the dimerization domain is folded into an isologous dimer stabilized by inter‐disulfide bonds between the two protomers, we also conducted SDS/PAGE under non‐reducing conditions to preserve any dimers. In the patient‐derived sample, there was a significant reduction in the level of RIβ in their dimeric form, as compared to the sample from the healthy individual (Fig. 2E,F).

### The RIβ‐L50R variant promotes C‐subunit translocation into the nucleus

The presence of RIβ‐L50R in the monomeric rather than the dimeric form, combined with the unchanged expression levels of the C‐subunit, led us to consider the subcellular localization of these subunits in the primary fibroblasts of both a healthy and a patient carrying the mutation in *PRKAR1B* (Fig. 2G–I). Cells were first treated with isoproterenol (ISO), a physiological activator of cAMP (Fig. 2G). This treatment led to nuclear accumulation of the C‐subunit in both healthy and patient‐derived cells; however, the increase was significantly greater in cells expressing the RIβ‐L50R variant. To further evaluate the impact of maximal cAMP elevation, cells were treated with a combination of ISO and isobutylmethylxanthine (IBMX), a phosphodiesterase inhibitor that prevents cAMP degradation (Fig. 2H). This combined treatment resulted in a similarly enhanced nuclear translocation of the C‐subunit in patient‐derived cells, consistent with the results observed under physiological stimulation. Representative images of these quantified results are shown in Fig. 2I, highlighting the increased nuclear localization of the C‐subunit in patient‐derived cells carrying the L50R variant following both ISO and ISO/IBMX treatments. These results suggest that in the presence of the RIβ‐L50R variant, the L50R:C heterodimer dissociates more readily when cAMP levels in cells increase.

### Controlling rapid cα‐subunit translocation into the nucleus by introducing the R211K replacement into the RIβ‐L50R:Cα heterodimer

**Fig. 3:**
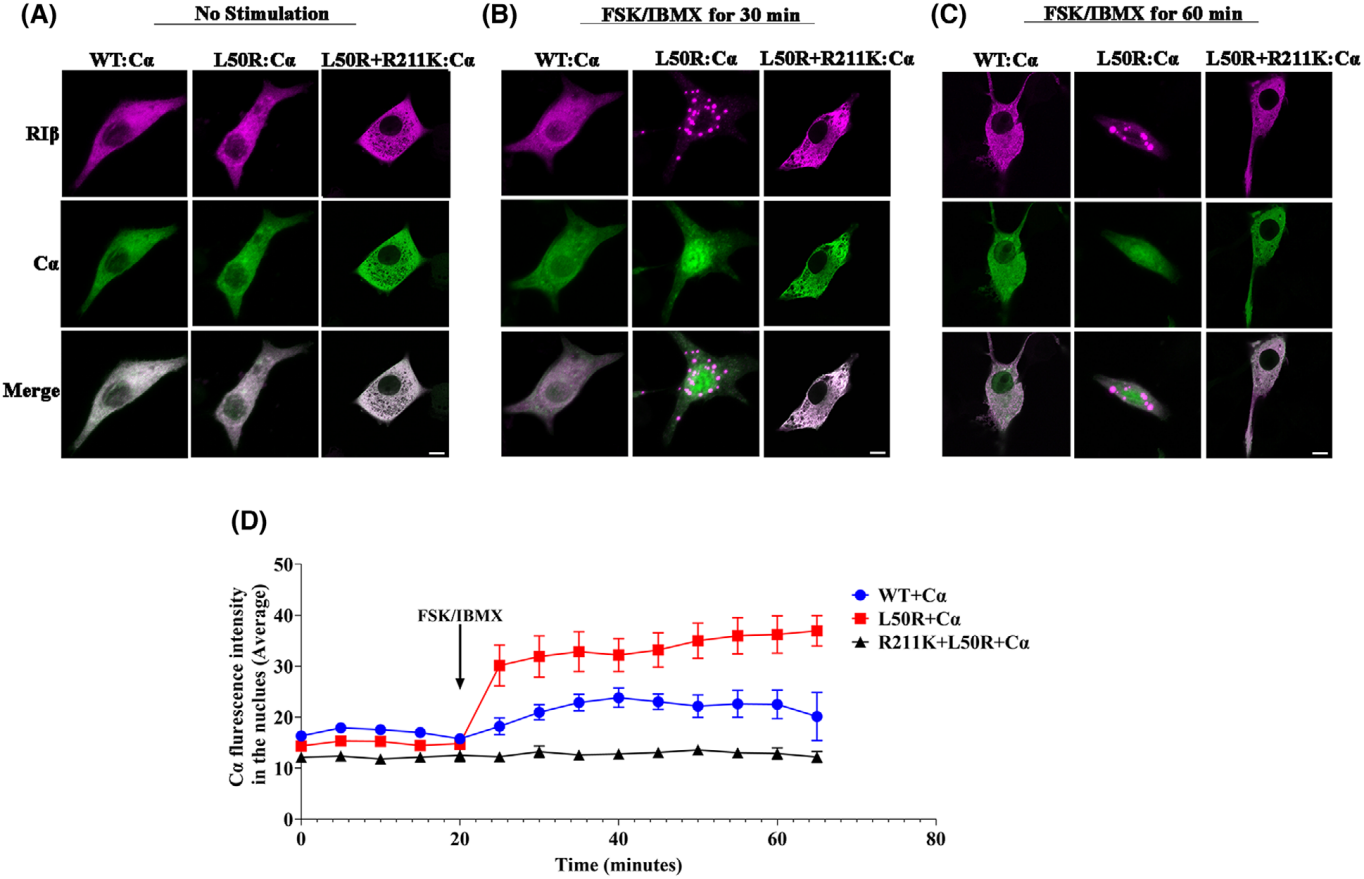
Controlling rapid Cα‐subunit translocation into the nucleus by introducing the R211K mutation into the RIβ‐L50R:Cα heterodimer. PC12 cells were co‐transfected to express mCerulean‐Cα along with mKO2‐ RIβ‐WT or mKO2‐ RIβ‐L50R or mKO2‐ RIβ‐L50R + R211K. Cells were imaged before treatment (A) and after treatment with 20 μm Forskolin (FSK) and 200 μm Isobutylmethylxanthine (IBMX) for 30 min (B) or 60 min (C) post fixation. Representative images from three independent experiments taken at 63X magnification are depicted. Scale bar: 10 μm. (D) Live‐cell imaging of PC12 cells co‐transfected to express mCerulean‐Cα along with RIβ‐WT or RIβ‐L50R or RIβ‐L50R + R211K. The cells were imaged every 5 min for 20 min before treatment and after treatment with 20 μm FSK and 200 μm IBMX as denoted in the graph (*n* = 5). The average fluorescence intensity of Cα in the nucleus was quantified in cells overexpressing the specified constructs. Data are presented as the mean ± standard error of the mean (SEM).

To further explore Cα translocation into the nucleus in response to cAMP‐induced RIβ:Cα complex dissociation, fluorescently labeled Cα and RIβ‐WT or mutant RIβ were co‐expressed in PC12 cells. Confocal microscopy was utilized to observe cellular dynamics before and after treatment with forskolin (FSK) and IBMX for 30 and 60 min, compounds which strongly elevate cAMP levels and cause dissociation of the RIβ:C heterodimer complex. An additional mutation that encodes an R211K replacement in the cAMP‐binding site A was introduced into the RIβ‐L50R‐expressing construct. This mutation is known to reduce R‐subunit sensitivity to cAMP [26]. PC12 cells co‐expressing RIβ‐WT or RIβ‐L50R or RIβ‐L50R‐R211K along with Cα were examined with or without cAMP level‐affecting stimulation (Fig. 3). An increase of Cα expression at the nucleus of cells overexpressing RIβ‐L50R was observed after 30 min of treatment compared to 60 min required for cells expressing the RIβ‐WT to exhibit Cα translocation. This indicates that the RIβ‐L50R:Cα complex dissociates more easily than the native complex (Fig. 3A–C). The introduction of the R211K mutation into the RIβ‐L50R mutant led to a protein that did not respond to elevated cAMP levels, and Cα failed to translocate into the nucleus (Fig. 3A–C). To further elucidate the sensitivity of the RIβ‐L50R:C complex to cAMP and to assess the effect of the R211K replacement on Cα translocation, we performed live‐cell imaging. Fluorescence intensity of the nucleus was calculated as an average intensity from images taken from five regions of the plate containing the labeled cells in the same time window. The graph in Fig. 3D demonstrates the similar average fluorescent intensity of Cα in the nucleus when co‐expressed with RIβ‐WT, RIβ‐L50R, or RIβ‐L50R‐R211K. However, upon treatment designed to elevate cAMP, a robust increase in Cα fluorescence intensity was measured upon RIβ‐L50R:Cα dissociation, as compared to that obtained upon RIβ‐WT:Cα dissociation. Introducing the R211K replacement into RIβ‐L50R further demonstrates the dependency of Cα translocation into the nucleus upon RIβ‐L50R:C dissociation. This live‐cell experiment is consistent with the images of fixed cells captured before and after 30 and 60 min of cAMP level‐affecting treatment (Fig. 3A–C), as both approaches demonstrated increased and rapid translocation of Cα into the nucleus upon elevation of cAMP levels in cells expressing the RIβ‐L50R mutant.

### RIβ‐L50R:Cα demonstrates altered holoenzyme dynamics in response to cAMP compared to the RIβ‐WT:Cα complex

**Fig. 4:**
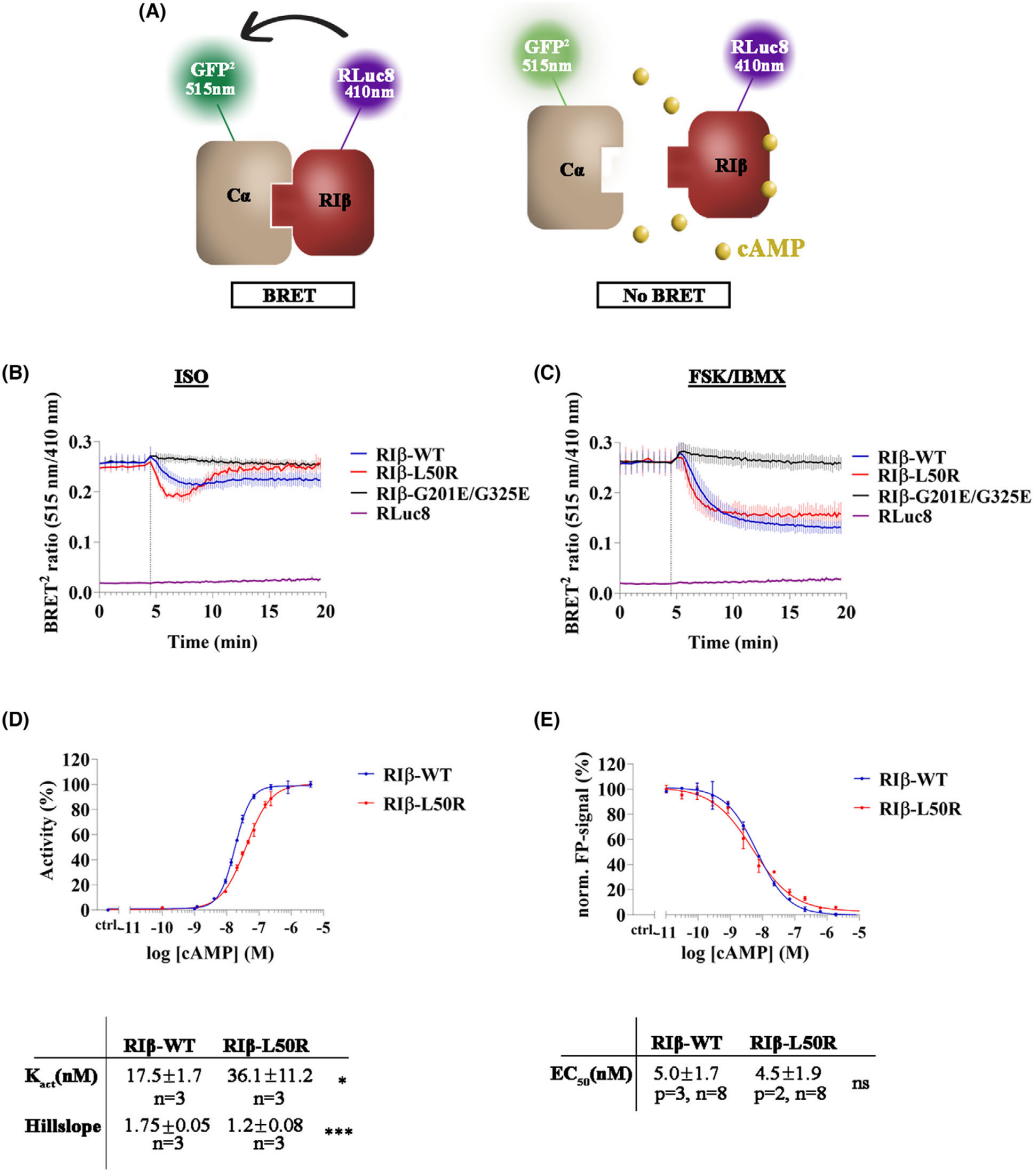
Altered PKA holoenzyme dynamics and loss of cooperativity induced by the RIβ‐L50R variant. (A) Illustration of PKA holoenzyme dynamics analyzed by the BRET^2^ system. GFP^2^‐tagged Cα and RLuc8‐tagged RIβ are denoted. cAMP is represented by yellow dots. (B, C) BRET^2^ signal profiles following stimulation of HEK293 cells with either 1 μm Isoproterenol (ISO) (B) or 50 μm Forskolin (FSK) and 100 μm Isobutylmethylxanthine (IBMX) (C). The BRET^2^ signal was monitored for 20 min. Data represent the BRET^2^ ratio calculated from the GFP^2^‐Cα signal (515 nm) divided by the RIβ‐RLuc8 signal (410 nm). RIβ‐G201E/G325E, unable to bind cAMP, served as control. An Rluc8 empty vector was used as a negative control for background luminescence. Each curve represents the mean of six replicates ± standard error of the mean (SEM). (D) cAMP‐mediated PKA holoenzyme activation was determined with a spectrophotometric kinase assay (representative curves). Normalized values for PKA activity were plotted against the logarithmic cAMP concentration. Activation constants (*K*_act_) and Hill slopes were determined by applying a sigmoidal dose–response fit (variable slope) and are summarized in the table below (*n* = number of measurements). The table shows the mean from three independent measurements in duplicate ± standard deviation (SD). Significance was tested with an unpaired *t*‐test (*P ≤ 0.05, ***P ≤ 0.001). (E) Fluorescence polarization assays measuring cAMP binding to His_7_‐RIβ‐WT or His_7_‐RIβ‐L50R (representative curves). Statistical analysis of the sigmoidal dose–response fits (variable slope) showed no significant differences (ns, not significant). All values are presented as the mean of multiple measurements ± SD, where “*p*” indicates the number of protein preparations and “*n*” indicates the number of measurements, each performed in duplicate. Significance was assessed using an unpaired t‐test following confirmation of normal distribution.

To further investigate RIβ‐L50R:Cα holoenzyme dynamics and to compare this heterodimer to the RIβ‐WT:Cα tetramer, we conducted a bioluminescence resonance energy transfer (BRET^2^) assay. In this assay, the R‐subunits were fused with RLuc8 while C‐subunits were fused with GFP^2^, as illustrated in Fig. 4A. Co‐transfection in HEK293 was performed to co‐express GFP^2^‐labeled Cα‐subunit together with RLuc8‐labeled RIβ‐WT, RIβ‐L50R, or RIβ‐G201E/G325E. The RIβ‐G201E/G325E was unable to bind cAMP and thus served as a negative control for cAMP sensitivity upon holoenzyme assembly [27, 28]. Cells overexpressing these vectors were treated with either FSK together with IBMX or with ISO, both resulting in increased intracellular cAMP levels. Cells overexpressing the RIβ‐WT:Cα complex dissociated upon FSK/IBMX treatment whereas cells overexpressing the RIβ‐G201E/G325E:Cα complex were unable to respond to the cAMP‐affecting stimulus, and as such, this holoenzyme did not dissociate. Notably, the BRET^2^ curve of the RIβ‐L50R:Cα complex resembled that of RIβ‐WT:Cα, indicating that holoenzyme dissociation in the presence of the RIβ‐L50R variant was similar to that of the native complex upon such stimulation (Fig. 4C). However, when exposed to 1 μm ISO, a condition that generates relatively physiological cAMP concentrations [29, 30, 31], a faster heterodimer dissociation was observed in the presence of the RIβ‐L50R variant compared to RIβ‐WT, as reflected by a more rapid decline in the BRET^2^ signal (Fig. 4B). [Correction added on 20 June 2025 after first online publication: “1 mm” has been corrected to “1 μm.”] This decrease was followed by a rapid re‐gaining of the BRET^2^ signal, resulting from a phosphodiesterase‐dependent cAMP degradation, indicating a faster holoenzyme reassociation of the RIβ‐L50R variant. These results suggest that the RIβ‐L50R mutant compromises the precise regulation of the heterodimer dynamics.

To quantify the effect of RIβ‐L50R substitution on PKA holoenzyme activation, we calculated the activation constants (*K*_act_) and Hill slope values for RIβ‐WT:Cα and RIβ‐L50R:Cα holoenzymes *in vitro* (Fig. 4D). A substantial difference was noted in the significantly lower Hill slope value calculated for the RIβ‐L50R holoenzyme, pointing to a loss of cooperativity in cAMP binding upon holoenzyme activation (Fig. 4D). While the Hill slope value for the RIβ‐WT:Cα holoenzyme indicated positive cooperativity, similar to a previously reported value for the isoform‐specific RIα:Cα holoenzyme, the RIβ‐L50R containing holoenzyme exhibited Hill slope values comparable to those of a monomeric RIα deletion mutant [32]. These findings further support the monomeric nature of RIβ‐L50R and emphasize the critical role of the D/D domain in promoting cooperativity. To explore whether the L50R variant directly affects cAMP binding to the RIβ‐subunit, we conducted a fluorescence polarization (FP) competition assay where cAMP competed with fluorescently labeled cAMP, 8‐Fluo‐cAMP (8‐(2‐[Fluoresceinyl] aminoethylthio) adenosine‐3′, 5′‐cyclic monophosphate) for binding to RIβ‐WT or RIβ‐L50R. As indicated in Fig. 4E, the EC_50_ values for cAMP were consistently in the low nanomolar range for both RIβ variants, implying that the L50R variant has no effect on the affinity of cAMP to the RIβ‐subunit, compared to the WT protein.

### Purified recombinant RIβ‐L50R does not dimerize and is prone to aggregation

**Fig. 5:**
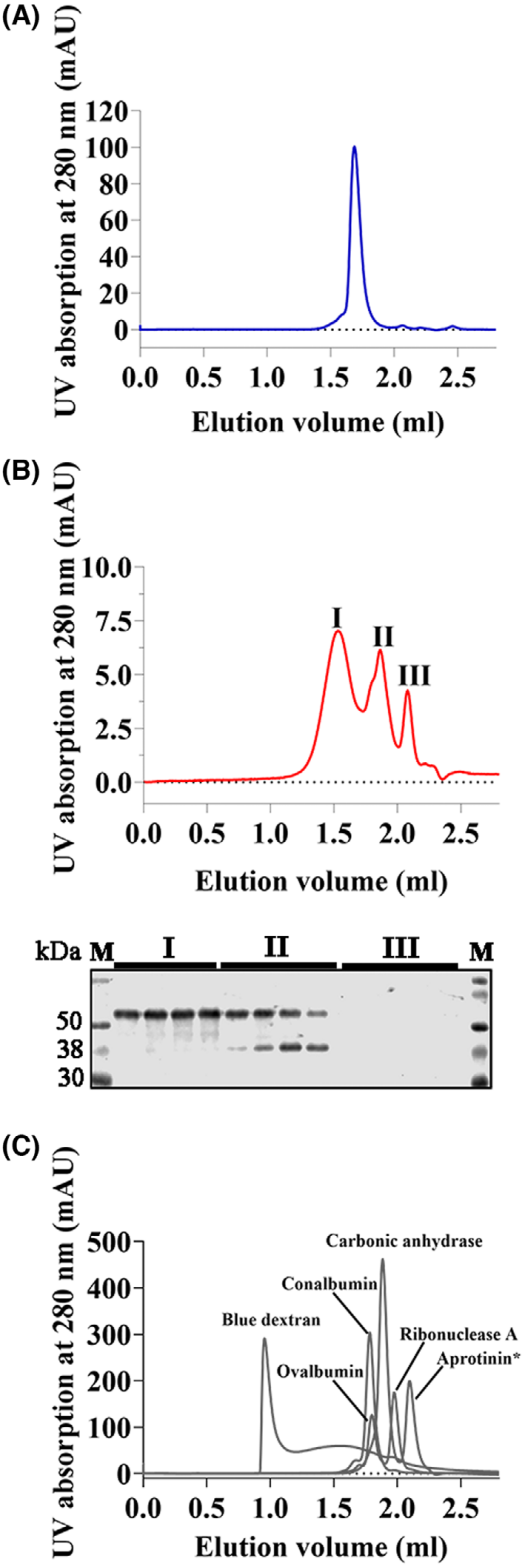
Disrupted dimerization and aggregation tendency of purified RIβ‐L50R proteins. Size‐exclusion chromatography profiles of (A) His_7_‐RIβ‐WT and (B) His_7_‐RIβ‐L50R. Western blot analysis of collected fractions from peak I, II, and III of the His7‐RIβ‐L50R using RIβ‐specific antibodies (B). Shown is a representative chromatogram from three independent measurements. (C) Chromatogram of standard proteins used to generate a calibration curve for calculation of Stokes' radii. Blue dextran was used for the determination of the void volume. *Aprotinin was excluded from the calculation of the standard curve.

To complement the cellular studies described above and to directly evaluate the ability of RIβ‐L50R to dimerize, we purified both full‐length RIβ‐WT and RIβ‐L50R and subjected them to size‐exclusion chromatography (Fig. 5A,B). RIβ‐WT eluted as in a single peak behaving as a stable dimer in solution, with a Stokes' radius of 45.3 Å. In contrast, RIβ‐L50R exhibited a distinct chromatographic profile containing multiple peaks, none of which corresponded to the expected size of a dimer (Fig. 5B). The significantly larger Stokes' radius of peak I (63.1 Å), as compared to the expected value associated with the dimer, suggests the presence of higher‐order multimers of this mutant RIβ protein. To further investigate the composition of the separated RIβ‐L50R species, we performed western blot analysis on the fractions collected (Fig. 5B). While peak I shows one band in the western blot analysis corresponding to the full‐length RIβ‐L50R protein, peak II reveals two different RIβ species. No RIβ could be detected in peak III. The first two fractions of peak II include a shoulder in the chromatogram which represents mainly full‐length RIβ‐L50R, while in the next two fractions a smaller band of RIβ‐L50R is more prominent. This shorter protein represents a truncated version of the RIβ‐L50R, most likely as a result of proteolytic degradation. A Stokes' radius of 25.7 Å resembles that of a RIα‐d1‐91 monomeric mutant previously described [32]. The Stokes' radius of full‐length RIβ‐L50R in peak II cannot be determined accurately; however, the elution volume (1.820 mL) of the shoulder of peak II corresponds to a monomeric RIβ protein rather than to a dimeric RIβ (1.687 mL, Fig. 5A). These data support the conclusion that in the RIβ‐L50R variant not only dimer formation is disrupted but also protein stability is weakened, and aggregation is promoted.

### The RIβ‐L50R variant shows enhanced cAMP‐induced dissociation of the RIβ‐L50R:Cα holoenzyme

**Fig. 6:**
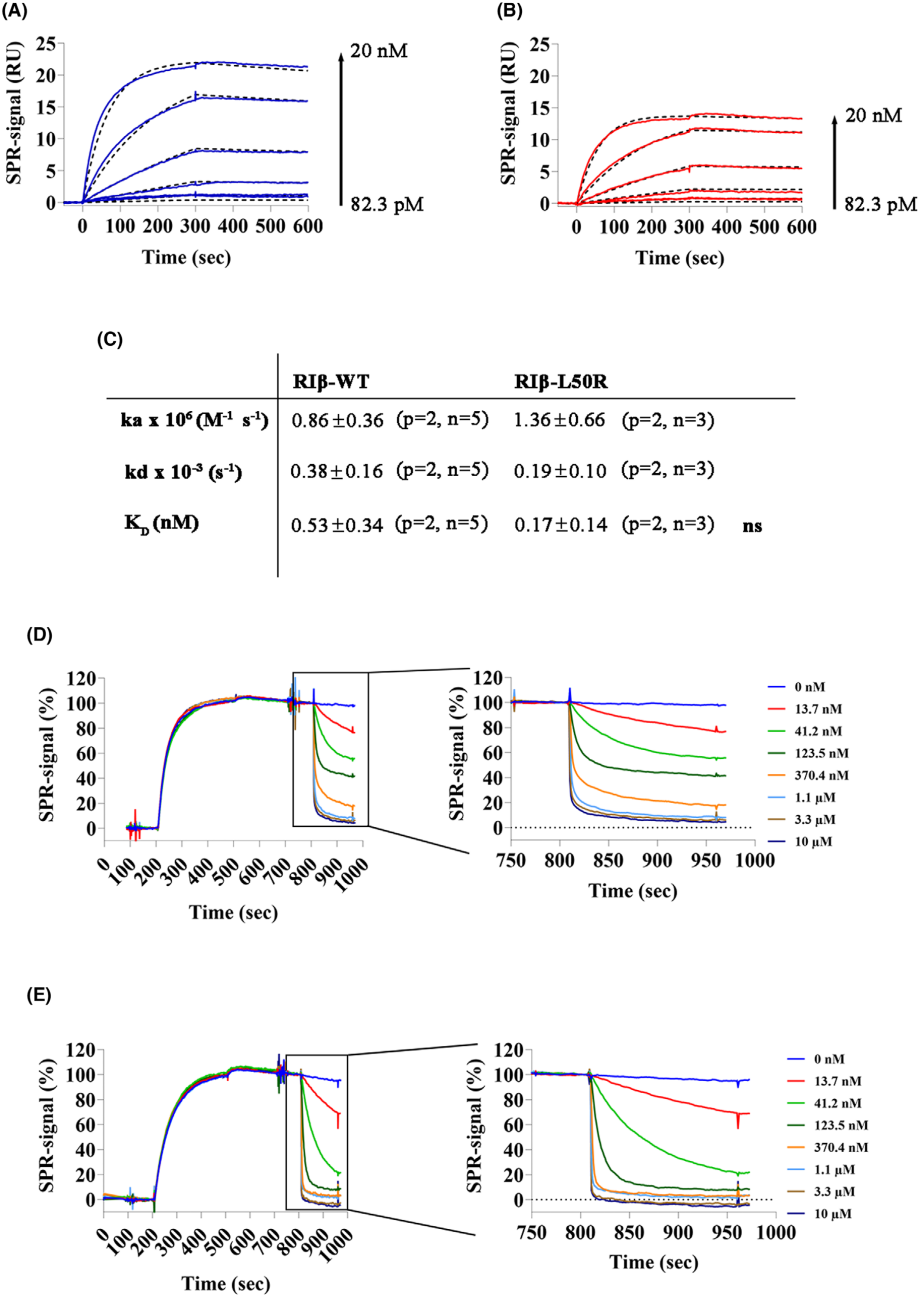
The RIβ‐L50R:Cα holoenzyme requires less cAMP to dissociate, despite high affinity binding to the Cα. (A, B) Binding kinetics of His_7_‐RIβ‐WT (A) and His_7_‐RIβ‐L50R (B) to immobilized FSS‐Cα‐subunit measured by surface plasmon resonance (SPR). Six different R‐subunit concentrations from 82.3 pm to 20 nm were used (dilution factor of 3). (C) Kinetic and equilibrium binding constants, determined using a 1 : 1 Langmuir binding fit model (Biacore T200 evaluation software 3.2) are summarized in the table. Values represent the mean ± standard deviation (SD) calculated from at least 3 independent sets of measurements, as denoted in the table (*p* = number of protein preparation, *n* = number of measurements). Significance was checked for with a Mann–Whitney test (ns, not significant). (D, E) cAMP‐induced dissociation of RIβ‐subunits from preformed holoenzymes determined by SPR. FSS‐Cα‐subunit was captured on a Strep‐Tactin‐coated CM5 sensor chip. 15 nm His_7_‐RIβ‐WT (D) or His_7_‐RIβ‐L50R (E) were injected to the FSS‐Cα‐subunit, reflected in an increase in the SPR signal. cAMP at different concentrations induced RIβ dissociation. Data were normalized at 190 s (0%) and 800 s (100%). Zoomed in sensograms highlight the differences in cAMP‐induced dissociation comparing RIβ‐WT:Cα and RIβ‐L50R:Cα. RU, response units.

Surface plasmon resonance (SPR) measurements were conducted to assess the impact of the L50R replacement on the RIβ:Cα affinity. FSS‐Cα was captured on a Strep‐Tactin coated sensor chip and RIβ‐WT or RIβ‐L50R, respectively, were injected in the presence of Mg^2+^ and ATP to determine the affinities between the respective R‐subunits and the C‐subunit (Fig. 6A,B). The calculated *K*_D_ values for the RIβ‐WT:Cα and RIβ‐L50R:Cα interactions were 0.53 ± 0.34 nm and 0.17 ± 0.14 nm, respectively, indicating sub‐nanomolar affinities between RIβ‐WT and RIβ‐L50R and the Cα‐subunit (Fig. 6C). These strong affinities are based in both cases on rapid association and slow dissociation phases (Fig. 6A,B).

To explore the effect of cAMP on the dissociation of R‐subunits from the immobilized Cα‐subunit, increasing concentrations of cAMP were added during the dissociation phase of the SPR assay (Fig. 6D,E). RIβ‐L50R dissociated at lower cAMP concentrations than those required by RIβ‐WT, suggesting a less stable mutant holoenzyme.

### Transcriptome analysis of patient primary fibroblasts

**Fig. 7:**
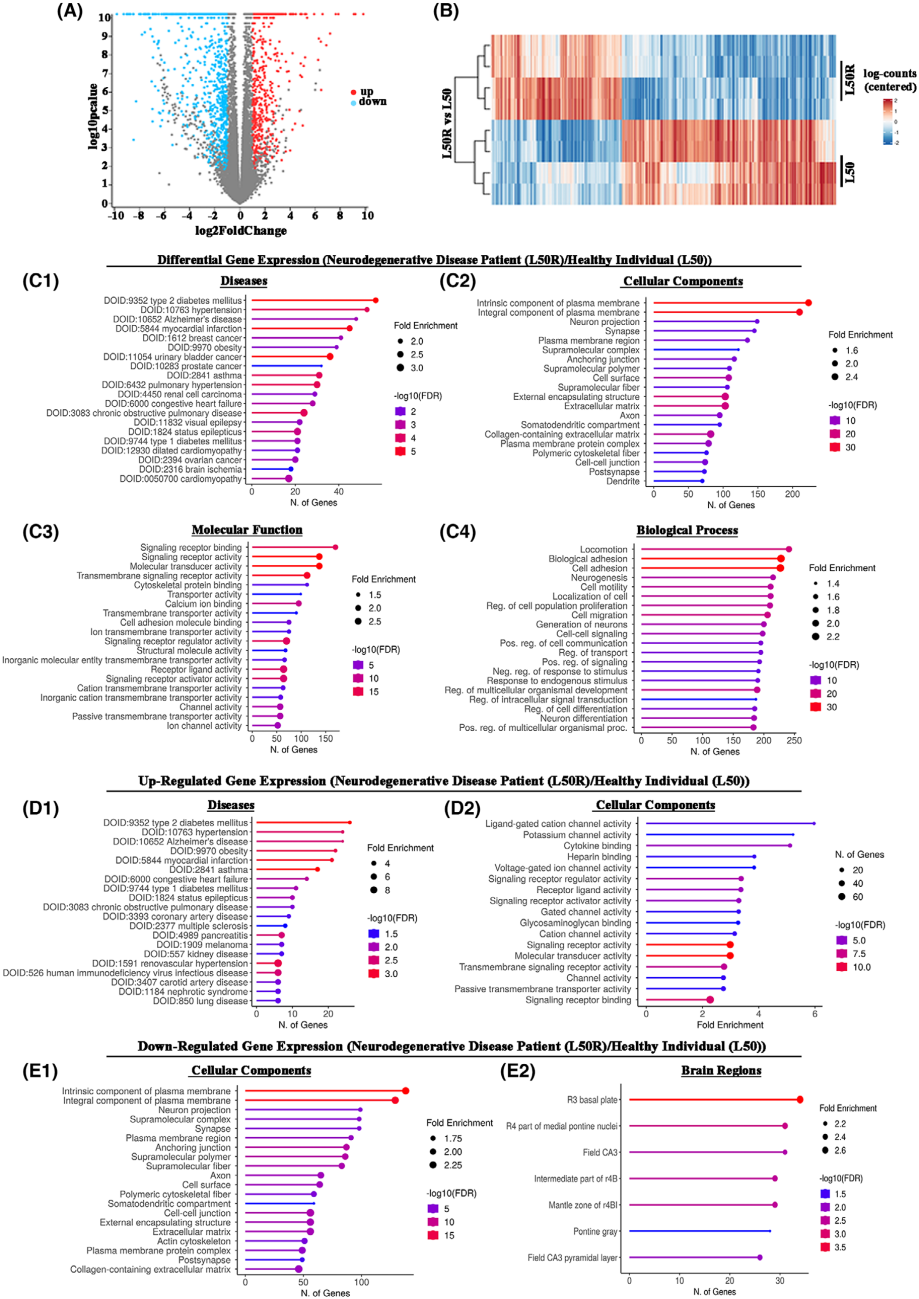
Differential gene expression analysis of primary human fibroblast cells. Analysis of the transcriptome changes in primary fibroblasts comparing a female patient expressing the RIβ‐L50R variant, diagnosed with a neurodegenerative disorder, and that of a healthy individual, each consisting of four independent cell samples. Analysis was conducted using ShinyGO: Gene Ontology Enrichment Analysis. (A) Volcano plot displaying differential expression. Blue color represents down‐regulated genes while red color represents up‐regulated genes in the affected individual (RIβ‐L50R), as compared to the healthy individual (RIβ‐L50). Genes with a log_2_foldchange ≥ 1 (corresponding to a multiple hypothesis‐adjusted *P*‐value (*P*adj) ≤ 0.05) are highlighted. (B) Heatmap illustrating gene expression patterns. Blue shading indicates down‐regulated genes, and red shading indicates up‐regulated genes in the RIβ‐L50R variant‐expressing subject, as compared to the RIβ‐L50 variant‐expressing subject. Darker shading indicates higher significance. (C) Differentially expressed genes that are up‐ and down‐regulated in the RIβ‐L50R variant‐expressing subject, as compared to the RIβ‐L50 variant‐expressing subject. Pathway classification analysis as denoted (C1‐4). (D) Pathway classification analysis of up‐regulated genes in the RIβ‐L50R variant‐expressing subject, as compared to the RIβ‐L50 healthy individual‐expressing subject (D1‐2). (E) Pathway classification analysis of down‐regulated genes in the RIβ‐L50R variant‐expressing subject, as compared to the RIβ‐L50 healthy individual‐expressing subject (E1‐2). The false discovery rate was calculated based on the nominal *P*‐value from the hypergeometric test. Fold‐enrichment is defined as the percentage of genes in a list belonging to a pathway, divided by the corresponding percentage in the background.

The biochemical and cellular data showing that PKA allosteric regulation is affected by the mutation resulting in RIβ‐L50R prompted us to perform transcriptome analysis so as to comprehensively understand the molecular consequences of unregulated PKA and its correlation with clinical symptoms. Analysis of the transcriptome from primary fibroblasts of a female patient diagnosed with a neurodegenerative disorder and expressing the RIβ‐L50R variant revealed significant changes in gene expression. This was compared to the transcriptome of fibroblasts from a healthy, age‐ and sex‐matched individual (Fig. 7). Of a total of 17 833 genes identified, 9002 were up‐regulated and 8831 were down‐regulated (ShinyGO v0.741). Only genes exhibiting log_2_foldchange ≥ 1, which were statistically significant at *P*adj ≤ 0.05, in the patient relative to the control, were further analyzed (Fig. 7). Disease‐based classification of the transcriptome revealed a gene expression signature associated with Alzheimer's disease (Fig. 7C1), as reflected by changes in the levels of transcripts for cellular components such as neuron projections, synapses, axons, and dendrites (Fig. 7C2). Notably, genes involved in locomotion function were significantly changed in the patient expressing the RIβ‐L50R mutant, consistent with the patient's clinical symptoms. Furthermore, we observed differential expression of key hub genes implicated in neuronal generation, neuronal survival, and growth, which correlated with MRI scans indicating pronounced brain atrophy, surpassing what is typical at the patient's age.

**Fig. 8:**
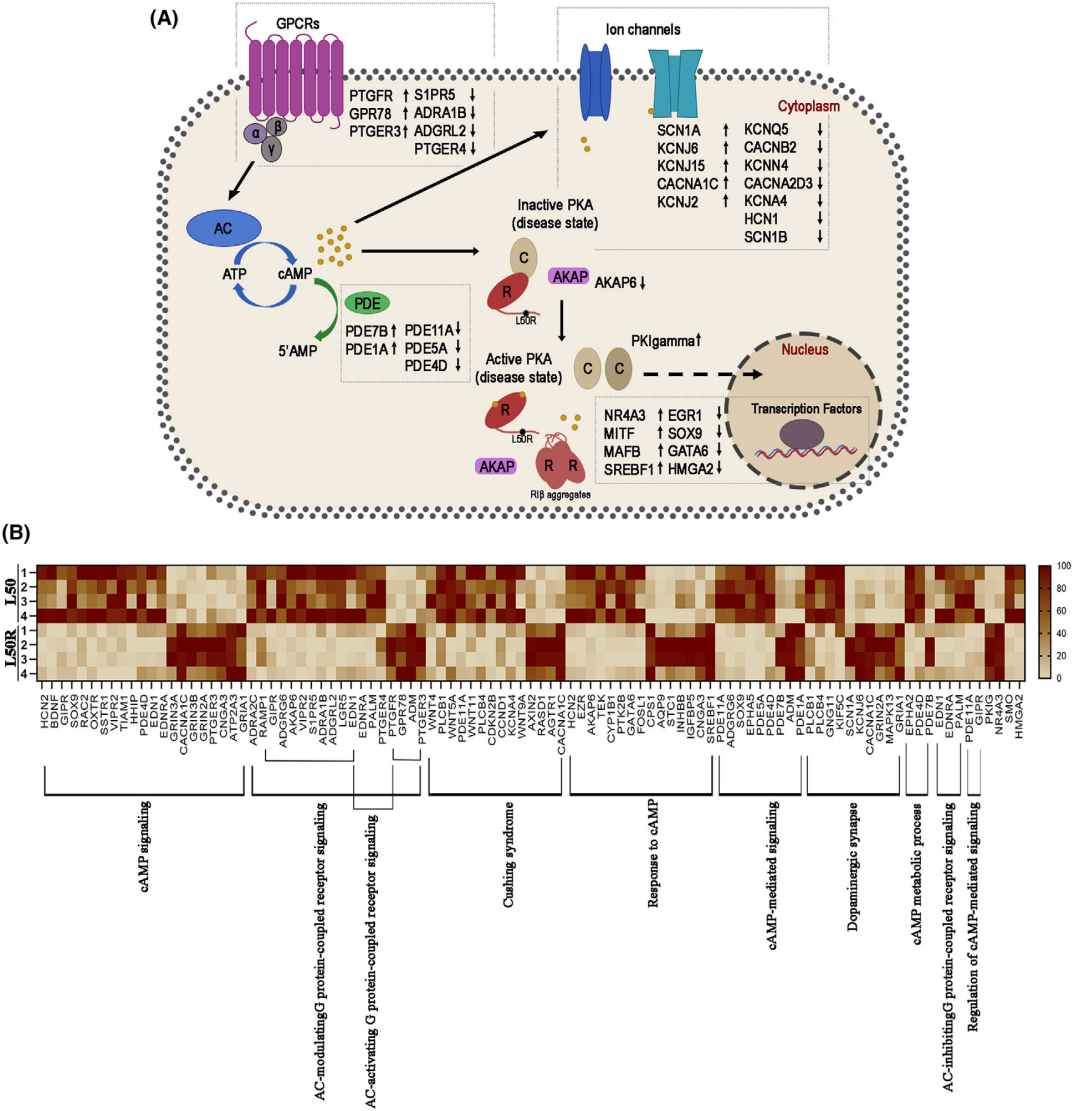
Differential gene expression of cAMP‐related pathway of genes in healthy individuals and RIβ L50R variant carriers. Enrichment analysis of primary fibroblasts from a patient carrying the RIβ‐L50R variant (denoted as L50R) compared to a healthy individual (L50), highlighting genes involved in cAMP‐related pathways. The analysis was conducted using Metascape, selecting genes with a log_2_foldchange ≥ 1 and an adjusted *P*‐value (*P*adj) ≤ 0.05. (A) Schematic representation of the cAMP signaling pathway with the RIβ‐L50R variant positioned at the center of the network. Arrows next to each gene indicate the direction of regulation: upward arrows denote up‐regulated genes, and downward arrows denote down‐regulated genes in L50R cells compared to L50. (B) Heatmap displaying the expression levels of cAMP‐related pathway genes across eight primary fibroblast samples—four from healthy individuals (L50) and four from the L50R variant patient. The heatmap highlights distinct gene expression patterns, with color gradients reflecting relative gene expression levels in each sample. AC, adenylyl cyclase; AKAP, A‐kinase anchoring protein; cAMP, cyclic adenosine monophosphate; GPCR, G protein‐coupled receptor; PDE, phosphodiesterase.

Enrichment analysis of differentially expressed genes, specifically those associated with cAMP‐related signaling pathways, was conducted using Metascape. Genes with a log_2_foldchange ≥ 1 and an adjusted *P*‐value (*P*adj) ≤ 0.05 were selected for further analysis. A schematic representation of the cAMP signaling pathway, with the RIβ‐L50R variant positioned at the center, is shown in Fig. 8A. Differentially expressed genes are mapped to their respective components within the pathway, with upward arrows indicating up‐regulated genes and downward arrows indicating down‐regulated genes in L50R cells compared to healthy controls.

To further visualize these changes, a heatmap of the expression levels of cAMP pathway‐related genes was generated for eight primary fibroblast samples—four from healthy individuals (L50) and four from the L50R patient (Fig. 8B). This heatmap demonstrates distinct gene expression patterns, highlighting consistent dysregulation of key genes involved in cAMP signaling, potentially contributing to disease phenotype.

## Discussion

In this study, we uncovered the molecular mechanisms underlying the clinical manifestations of neurodegeneration in patients expressing the RIβ‐L50R variant, revealing disrupted PKA holoenzyme assembly and uncontrolled allosteric regulation. Utilizing patient‐derived cells, as well as direct measurements of purified proteins, we shed light on the critical position of RIβ‐L50 in facilitating the dimer formation of the regulatory subunit, crucial for the precise regulation dynamics of the PKA holoenzyme.

Exploration of *PRKARIB* variants via database screening uncovered a clustering of mutations predominantly within the cAMP‐binding domain CNB‐B, with significantly fewer mutations in CNB‐A and the D/D domain. This pattern highlights the evolutionary conservation of CNB‐A and the D/D domain. The CNB‐A domain serves as a central hub for allosteric communication between R and C, stabilizing the holoenzyme and enabling efficient cAMP‐mediated activation [33]. Similarly, the D/D domain's conservation reflects its critical role in dimerization and anchoring, ensuring proper holoenzyme assembly and regulation.

Our focused analysis on the L50R *PRKARIB* variant, the only known PKA regulatory subunit variant confirmed to disrupt dimer formation, not only unraveled the dynamic properties of PKA assembly and allosteric regulation but also provided insights into the functional consequences leading to neurodegenerative disease. Although other pathogenic RIβ variants, such as the I40V, R68Q, and A67V mutants, presented replacements of residues localized within the D/D domain, they did not disrupt dimerization and thus were not the primary focus of our study.

Integration of structural analysis results with those obtained in biochemical studies employing purified proteins, and cellular studies involving cells overexpressing targeted fluorescent proteins, as well as patient‐derived cells, consistently indicated that the mutation leading to the appearance of the RIβ‐L50R variant disrupted dimer formation and rendered the protein prone to aggregation. Size‐exclusion chromatography revealed distinct differences between recombinant RIβ‐WT and RIβ‐L50R proteins, with RIβ‐WT exhibiting a stable dimeric structure while RIβ‐L50R displayed a high tendency to form multi‐oligomers. CD spectroscopy analysis corroborated these findings, demonstrating alteration in secondary structure and stability of the protein, in line with predictions from the *in‐silico* modeling. Notably, the CD spectroscopy results for the monomeric L50R mutant revealed a substantial reduction in alpha‐helical content, indicating that dimerization and folding might be coupled.

In cells, RIβ‐L50R aggregates were detected in the insoluble fraction. Relying on the approaches listed above, we were able to conclude that such aggregation resulted from structural perturbation and misfolding of the D/D domain. Consequently, the docking site for AKAPs within this domain failed to appear, leading to the mutant protein being unable to bind dAKAP1. This emphasizes the critical importance of proper folding of this PKA R‐subunit domain for assembly of macromolecular complexes.

The molecular mechanism elucidated here for a PKA dependent neurodegenerative disease in which protein aggregation results from disrupted homodimerization sheds light on a potentially common but under‐appreciated mechanism shared by several neurodegenerative diseases. Mutations in the *DJ‐1* gene associated with autosomal recessive early‐onset Parkinsonism result in destabilized homodimerization [34, 35]. The crystal structure of human DJ‐1 demonstrated the protein to exist as a stable homodimer. The mutation leading to L166P replacement in DJ‐1, identified as causal in patients with Parkinson's disease, produces an unstable form of the protein that forms higher‐order oligomers and is degraded by the proteasome [36]. Similarly, mutations in SOD1 associated with amyotrophic lateral sclerosis (ALS) share a similar molecular mechanism of dimer destabilization [37, 38, 39]. This emphasizes the broader significance of disrupted homodimerization in protein aggregation and disease pathology.

**Fig. 9:**
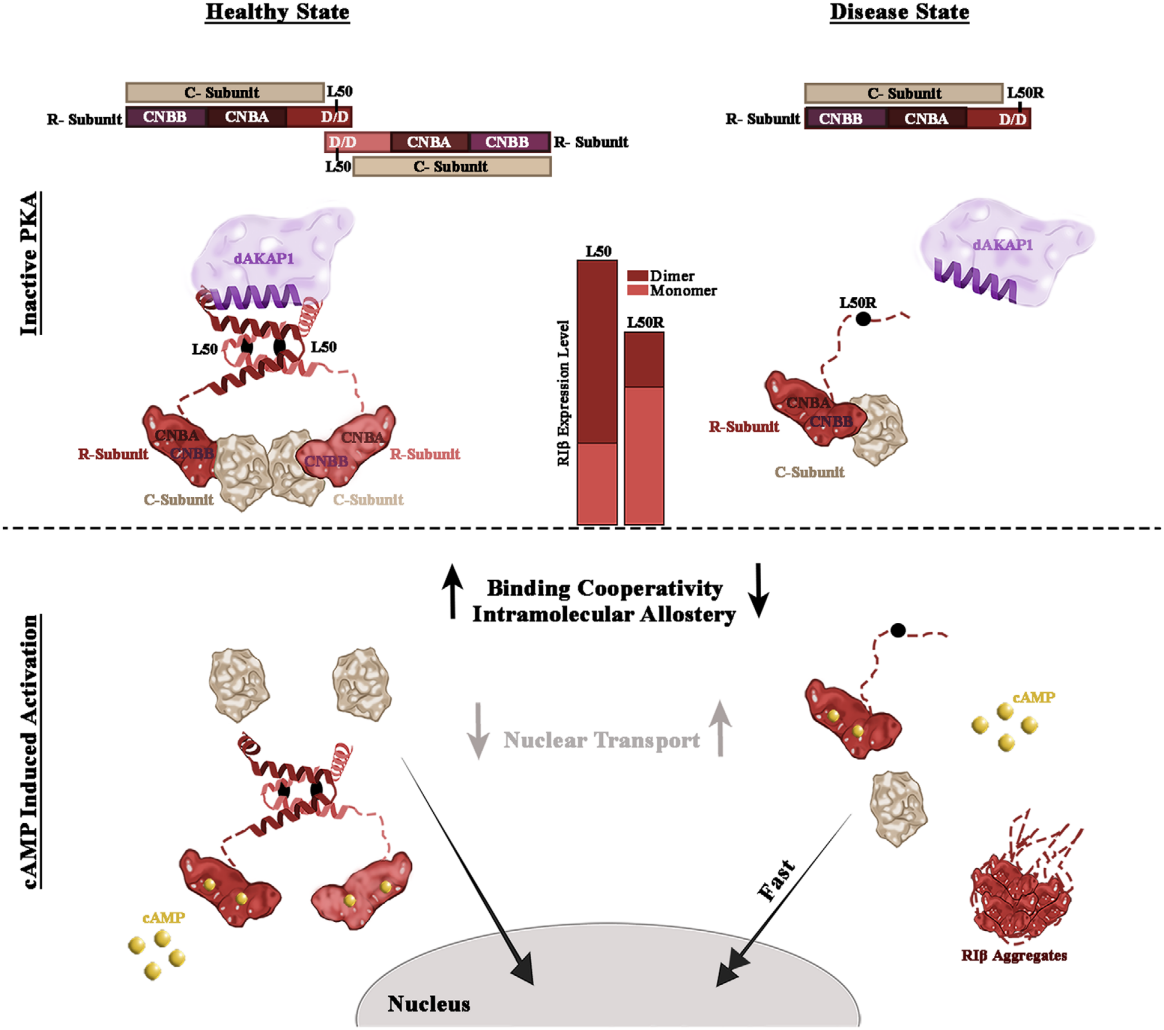
Illustration of PKA holoenzyme assembly and dynamics in the healthy and disease state before and in response to cAMP stimulation. Upper panel depicting the inactive state. In the healthy state, the PKA holoenzyme is a dimer of two RIβ:C heterodimers. Homodimerization provides a binding site for AKAPs. In the disease state, PKA exists as a RIβ:C heterodimer, where the L50R mutant perturbs homodimerization and AKAP binding. The bars in the illustration represent RIβ expression levels and the ratio between monomers and dimers. The lower panel depicts PKA dynamics upon cAMP‐induced activation. In the healthy state, the RIβ dimer dissociates from the C‐subunit, allowing its translocation into the nucleus. In the disease state, the RIβ‐L50R:C heterodimer dissociates faster, exhibits reduced cooperativity, leads to RIβ aggregation, and promotes faster translocation of the C‐subunit into the nucleus. cAMP, cyclic adenosine monophosphate; CNB, cyclic nucleotide‐binding domain; D/D, dimerization and docking domain.

We previously solved the crystal structure of the RIβ holoenzyme as a dimer of two heterodimers composed of a regulatory and catalytic subunit [40]. In the absence of a folded dimerization domain, RIβ‐L50R can interact with the catalytic subunit to form a heterodimer instead of a dimer of heterodimers (Fig. 9). We have shown that the interaction of RIβ‐L50R with a catalytic subunit protects the former from aggregation [25]. In the present study, we sought to dissect the allosteric network of the RIβ holoenzyme and determine how the mutation leading to the expression of the L50R variant impacts PKA holoenzyme dynamics. We showed that RIβ‐L50R binds with high affinity to the C‐subunit, as measured by SPR. The retained high affinity of the RIα regulatory subunit that lacks the N‐terminal D/D domain (RIα‐Δ1‐91) for the C‐subunit was shown earlier [32]. Our results confirmed that the RIβ:Cα interactions for one heterodimer are remarkably similar for the different RI isoforms [16] and that the differences between the isoforms occur at the tetrameric level. The presence of RIβ‐L50R in its monomeric, rather than its dimeric form, led us to investigate the subcellular localization of RIβ‐L50R and the C‐subunit in primary fibroblasts from both a healthy individual and a patient with the mutation. While the localization of RIβ remained unchanged in the presence of the mutation, an increase in C‐subunit expression within the nucleus was evident in the patient expressing the mutant protein under physiological cAMP stimulation. This increase was also replicated in PC12 cells treated with cAMP stimulators, suggesting that the RIβ‐L50R:C heterodimer dissociates easier than the RIβ_2_:C_2_ holoenzyme in the presence of cAMP within the cell. This is in line with the BRET^2^ results reported here.

In contrast to the higher cAMP sensitivity of the RIβ‐L50R:C heterodimer demonstrated in cells and in SPR measurements, the *in vitro* activation assay indicates a slightly higher *K*_act_ value for the L50R compared to the wildtype holoenzyme, with (*P*‐value of *P* = 0.0475). Oligomeric forms of degradation products of L50R that do not form holoenzyme may still be capable of binding cAMP, thereby shifting the respective *K*_act_ to higher values. Our results, however, clearly show that the L50R holoenzyme displayed reduced cooperativity in activation, as compared to the wildtype complex, evident from a significantly lower Hill slope value (*P* = 0.0004). These results emphasize the role of the RIβ N‐terminal domain in maintaining cooperativity of the PKA holoenzyme and suggest that the L50R mutant compromises the precise regulation of heterodimer dynamics. Based on the findings of this study, we propose that decreasing the affinity of RIβ‐L50R to cAMP could offer a promising strategy to restore regulation over the reduced allosteric mechanisms caused by the associated mutation. This approach thus presents a potential avenue for therapeutic intervention.

Our comprehensive investigation of the dynamics of the RIβ‐L50R:C heterodimer, ranging from *in vitro* analysis of the purified proteins to visualization of complex assembly and dynamics in cells derived from a patient affected by a neurodegenerative disease, helped unravel the intricate interplay between PKA holoenzyme structure in the presence of the mutant, disrupted assembly and dynamics, and the onset of neurodegenerative phenotypes. These phenotypes are reflected by a unique pattern of gene expression specific to the disease (i.e., NLPD‐PKA), which shares commonalities with changes observed in other neurodegenerative diseases. For instance, alternations in the expression of key hub genes associated with neuronal degeneration, growth, and survival are indicative of the severe brain atrophy observed in MRI scans of affected patients expressing the RIβ‐L50R variant. This pattern of gene dysregulation mirrors findings seen in other neurodegenerative diseases, such as Alzheimer's disease, where similar alternations in gene expression profiles have been reported [41]. Furthermore, the observed dysregulation of cellular components, including neuron projections, synapses, axons and dendrites in the patient expressing the RIβ‐L50R variant is consistent with the pathological changes seen in other neurodegenerative diseases. Examples of such diseases include Parkinson's disease, where alternation in neuronal projections and synapses contribute to motor impairments [42], Huntington's disease, characterized by abnormalities in axonal transport and dendritic spine densities resulting in cognitive dysfunction [43], and ALS, where abnormalities in axonal transport and axonopathy contribute to progressive muscle weakness [44].

These changes reflect synaptic disruption and neuronal loss, as well as disrupted neuronal circuits, all of which are common features observed in various neurodegenerative conditions. Given that PKA dysfunction contributes to the pathogenesis of various neurodegenerative diseases [8, 9, 10, 11, 12, 13], establishing a direct link between unregulated PKA allostery and assembly and the products of genes associated with neurodegeneration will help identify therapeutic targets for maintaining PKA function.

## Materials and methods

### Subjects

The study methodologies conformed to the standards set by the Declaration of Helsinki (1984) and its subsequent revisions. The research was approved by the Medical Ethical Committee of the Erasmus Medical Center Rotterdam (MEC‐2009‐401).

Written informed consent for the use of clinical data and tissue samples (Fibroblasts) was obtained from all participants or their legal representatives.

The study included patients diagnosed with NLPD‐PKA, who were followed for diagnostic evaluation and fibroblast collection between January 2020 and May 2021.

### Cell lines and cell culture

Primary human fibroblast cells, rat pheochromocytoma (PC12) cells, and human embryonic kidney 293 (HEK293 RRID:CVCL_0045) cells were maintained in high glucose Dulbecco's modified Eagle's medium (DMEM) (Biological Industries) supplemented with 10% fetal bovine serum (Biological Industries, Kibbutz Beit Haemek, Israel), 2 mm L‐glutamine (Sartorius, Göttingen, Lower Saxony, Germany) and 5% penicillin–streptomycin (Biological Industries) at 37 °C in an atmosphere of 5% CO_2_. All cell lines were negative for mycoplasma. *E. coli* Δcya TP2000 cells were kindly provided by Choel Kim (Baylor College of Medicine, Houston, TX, USA). BRET^2^ measurements were performed with eukaryotic HEK293 cells (RRID:CVCL_0045, Leibniz‐Institut DSMZ).

### Mutagenesis and plasmids

Single‐site mutations that led to the replacement of the indicated amino acids at the following positions I40V, L50R, A67V, R68Q, and R211K were introduced into the mKO2‐RIβ plasmid using a site‐directed mutagenesis kit (New England BioLabs, Ipswich, MA, USA). To generate recombinant human His‐tagged RIβ‐WT and ‐L50R proteins, a pQTEV vector containing a sequence for an N‐terminal 7x‐polyhistidin‐tag was used. All constructs were confirmed by sequencing.

### Antibodies

Primary antibodies: Sheep anti‐PKA RIβ antibodies [R&D Systems catalog # AF4177 (RRID: AB_2284184), Minneapolis, MI, USA] were diluted 1 : 2500 for western blot (WB), and 1 : 100 for immunohistochemistry (IHC). Mouse anti‐PKAc monoclonal antibodies BD Biosciences catalog # 610981 (RRID: AB_398294) were diluted 1 : 4000 for WB, 1 : 50 for IHC. Rabbit anti‐GAPDH Abcam Catalog # ab9485 (RRID: AB_307275, Cambridge, UK) dilution 1 : 2500 for WB. Rabbit anti‐Topoisomerase I Abcam Catalog # ab109374 (RRID: AB_10861978) dilution 1 : 1000 for WB. Rabbit Anti‐AKAP1 Cell signaling Catalog #CST‐5203 (RRID: AB_10828202) dilution 1 : 1000 for WB, 1 : 100 for IHC. The WB primary antibodies were prepared in 1% filtered BSA in PBST solution, and the IHC primary antibodies were prepared in 1 : 10 blocking solution [10% Normal Donkey Serum (Jackson ImmunoResearch, West Grove, PA, USA) + 0.1% filtered BSA] in PBST. Rabbit Anti‐PKA‐RIβ (ABM catalog #Y051648, Richmond, British Columbia, Canada) diluted 1 : 2000 in TBS‐T buffer (20 mm Tris, pH 7.5, 140 mm NaCl and 0.1% (v/v) Tween 20) supplemented with 1–2% (w/v) milk powder was used as the primary antibody for the WB of the analytical gel filtration fractions. Secondary antibodies: Donkey Anti‐Sheep IgG, HRP Conjugated (Abcam) (RRID: AB_955452), Goat Anti‐Rabbit IgG, HRP Conjugated (Abcam) (RRID: AB_955447), Rabbit to Mouse IgG, HRP Conjugated (Abcam) (RRID: AB_955440), Donkey Anti‐Sheep IgG (Alexa Fluor 647) Abcam Catalog # ab150179, Goat Anti‐Rabbit IgG (L + H) (Alexa Fluor 488) Invitrogen Catalog # A‐11934 (Waltham, MA, USA). All HRP‐secondary antibodies were used at 1 : 10 000 dilution and all fluorescent‐secondary antibodies were used at 1 : 250 dilution. IRDye 800CW Donkey Anti‐Rabbit (LI‐COR Biosciences, Lincoln, NE, USA) (catalog # 926‐32213) diluted 1 : 15 000 in TBS‐T buffer with 1–2% (w/v) milk powder was used as a secondary antibody for the WB of the analytical gel filtration fractions.

### Transient transfection

PC12 cells were grown in 6‐well plates for WB and in 24‐well plates for immunofluorescence (IF). After 24 h of incubation, the cells were transfected with 2 m Calcium Chloride and DNA (0.5 μg for 24 well or 5 μg of 6 well plates). The solution was added to HEPES‐buffered saline solution with the same volume and incubated for 15 min at room temperature. After incubation, the transfection solution was gently added to the cell media and the cells were lysed for WB or fixed for IF after 48 h.

For time‐dependent BRET^2^ measurements 2 × 10^4^ HEK293 cells per well were seeded on a 96‐well microtiter plate (Nunclon™ Delta Surface, Thermo Scientific, Waltham, MA, USA) and grown for 24 h at 37 °C and 6% CO_2_. DMEM (Capricorn Scientific, Ebsdorfergrund, Hessen, Germany) with 10% FBS (v/v) (Capricorn Scientific) was used as the culture medium. After 24 h, 0.05 μg DNA of C‐terminally RLuc8 [45] tagged WT or L50R RIβ‐subunits and 0.05 μg DNA of N‐terminally GFP^2^ tagged C‐subunits were co‐transfected per well as previously described. Cationic polyethyleneimine (PEI, Polysciences GmbH, Hirschberg an der Bergstrasse, Baden‐Württemberg, Germany) solution was used to improve transfection [46]. Proteins were expressed for 48 h at 37 °C and 6% CO_2_. 24 h after transfection, medium was exchanged with fresh DMEM medium including 10% (v/v) FBS (Capricorn Scientific).

### Cell fractionation and WB

Primary human fibroblasts were grown to approximately 70% confluence in 10 mm plates and incubated for 48 h, or 3 × 10^5^ PC12 cells were grown in a 6‐well plate for 24 h, transfected, and incubated for 48 h. Both cell types were washed twice in cold PBS (Biological Industries), suspended in lysis buffer containing 50 mm Tris/HCl pH 7.5, 0.1% Triton X‐100, 150 mm NaCl, 1 mm EGTA, 1% NP‐40, 0.25% sodium deoxycholate, 1 mm PMSF, and a cocktail of protease inhibitors (P8340 Sigma‐Aldrich, St. Louis, MI, USA) (diluted 1 : 100) for primary fibroblasts, or in lysis buffer containing 50 mm Tris/HCl pH7.4, 150 mm NaCl, 1 mm EDTA, 0.1% Triton X‐100, 1% NP‐40, 10 mm NaF, 1 mm Na_3_VO_4_, phosphatase inhibitors, and protease inhibitors for PC12 cells, and incubated on ice for 15 min. The lysates were centrifuged at 21 300 *g* and 4 °C for 30 min. for PC12 cells, after centrifugation, the supernatant was collected as the soluble fraction, and the pellet was washed twice in cold PBS, resuspended in lysis buffer containing 6 m urea, and sonicated. Sample buffer X5 with or without SDS and β‐Mercaptoethanol (for non‐reduced conditions) was added, and the samples under reducing conditions were boiled for 4 min. Protein lysates were resolved by SDS/PAGE and transferred to PVDF membranes (Bio‐Rad) using a Trans Blot Turbo RTA midi transfer kit (Bio‐Rad, Hercules, CA, USA). The PVDF membranes were blocked with 5% Bovine serum albumin (BSA) in PBST (0.1% Tween 20) for 1 h at room temperature, and subsequently incubated overnight at 4 °C with primary antibodies. After incubation, the PVDF membranes were washed four times with PBST for 10 min. secondary antibodies with HRP were incubated for 1 h. After four washes with PBST, the PVDF membranes were detected using ECL.

### Immunohistochemistry and immunofluorescence

Primary fibroblasts were grown on 13 mm coverslips in 24‐well plates to approximately 70% confluence and incubated for 48 h. After treatment, the cells were washed twice in PBS and fixed for 15 min in 4% paraformaldehyde. After fixation, the cells were washed five times with PBS and permeabilized and blocked in PBS containing 0.5% Triton‐X100 and 1% BSA for 30 min. After blocking, the cells were immunostained overnight at 4 °C with primary antibodies. After this time, the cells were washed three times in PBS and incubated with a secondary antibody for 1 h at room temperature and washed twice with PBS. Nuclei were counterstained with Hoechst (Invitrogen) for 5 min. For PC12 cells, 0.6 × 10^5^ PC12 cells were grown on a 13 mm coverslip in a 24‐well plate for 24 h, co‐transfected, and incubated for 48 h. After treatment, the cells were washed twice in PBS and fixed for 15 min in 4% paraformaldehyde, then washed three times with PBS and stained with Hoechst for 5 min. The slides were mounted on cover glass with Gelvatol and imaged on a Zeiss LSM 780 confocal microscope and Leica STED live imaging microscope.

### PKA RIβ dimerization/docking domain expression and purification

The Dimerization/Docking (D/D) domain of wildtype PKA‐RIβ was produced in *Escherichia coli* BL21(DE3) pLysS grown at 37 °C until the optical density at 600 nm reached 0.6. Expression was induced with 0.5 mm isopropyl β‐D‐1‐thiogalactopyranoside (IPTG) at 18 °C overnight. Cells were harvested by centrifugation (5000 ***g***, 11 min, 4 °C), resuspended in lysis buffer (50 mm Tris, 500 mm NaCl, 1 mm DTT, pH 7.5, with protease inhibitors), and lysed using a microfluidizer at 10 000 psi. Cell debris was removed by centrifugation (15 000 ***g***, 45 min, 4 °C), and soluble proteins were precipitated with 45% ammonium sulfate (1 h, 4 °C). The precipitate was collected by centrifugation, redissolved in lysis buffer, and clarified again by centrifugation (16 000 ***g***, 10 min, 4 °C). The resulting supernatant was incubated overnight at 4 °C with nickel‐coupled agarose resin pre‐equilibrated in lysis buffer. Bound protein was eluted with buffer containing 500 mm imidazole, unfolded in 8 m urea with 5 mm DTT for 30 min at room temperature, and refolded by gradual dialysis into gel filtration buffer (50 mm Tris, 200 mm NaCl, pH 7.5) over 24 h at 4 °C. The refolded protein underwent size‐exclusion chromatography (HiLoad 16/600 Superdex 200 pg, Cytiva, FPLC Bio‐Rad, Marlborough, MA, USA) to remove aggregates. Fractions matching the expected dimer were pooled, verified for dimer stability via a second chromatography run, and stored at −80 °C in gel filtration buffer with 25% glycerol.

The L50R mutant of the D/D domain was expressed similarly but formed predominantly inclusion bodies. Inclusion bodies were isolated by homogenization in extraction buffer (8 m urea, 50 mm Tris, 500 mm NaCl, 5 mm DTT, pH 7.5, with protease inhibitors) for 30 min at room temperature, followed by centrifugation (15 000 ***g***, 15 min). The supernatant containing solubilized L50R protein was refolded via stepwise dialysis into gel filtration buffer at 4 °C. Subsequently, the protein was purified using nickel‐agarose resin, eluted with buffer containing 500 mm imidazole, and subjected to size‐exclusion chromatography for final purification. Purified L50R protein was stored in gel filtration buffer with 25% glycerol at −80 °C.

### Secondary structure analysis of the PKA RIβ D/D domain

Circular dichroism (CD) was used to evaluate the secondary structure of the D/D domain of PKA RIβ wildtype and L50R. Experiments were performed using a Jasco J‐715 spectrometer, and all signals were corrected for buffer effects (50 mm Tris, 200 mm NaCl, pH 7.5). The data was collected using 15 μm of protein, with the following instrument parameters: continuous mode from 200 to 260 nm, pathlength = 0.1 cm, sampling rate 10 nm·min^−1^, and a bandwidth of 10.0 nm. 13 and 9 scans were averaged for wildtype and L50R, respectively. Data analysis to estimate secondary structure was performed using BestSel [47, 48, 49, 50, 51].

### cAMP stimulation

48 h after PC12 cells or primary fibroblasts transfection, the media was replaced with serum‐free DMEM and incubated for 1 h. Then pharmacological agents were introduced for primary fibroblasts: 1 μm Isoproterenol (ISO, Abcam) or 200 μm Isobutylmethylxanthine (IBMX, Sigma‐Aldrich) and 1 μm ISO was gently added to the cell media for 30 min and fixed for the IF assay. PC12 cells were treated with a cocktail of 20 μm Forskolin (FSK, Abcam) and 200 μm IBMX (Sigma‐Aldrich) and incubated for 30 min and 1 h before fixation for the IF assay.

### Live‐cell imaging

3 × 10^5^ PC12 cells were grown on a 35 mm glass bottom dish. After 24 h, the cells were co‐transfected and allowed to incubate for 48 h. One hour prior to the imaging, the medium was replaced with serum‐free DMEM. The cells were imaged every 5 min for 20 min at five different positions. Then, the cells were treated with 20 μm FSK and 200 μm IBMX, and imaging of the same positions continued at 5 min intervals. Image quantification was conducted using image j software (National Institutes of Health, Bethesda, MD, USA).

### Image processing and relative quantification of laser intensity in the nucleus

A customized code written in Java for the fiji (Version 1.54g 18 October 2023, Laboratory for Optical and Computational Instrumentation (LOCI), University of Wisconsin‐Madison, Madison, WI, USA). Image processing platform was used to relatively quantify the intensity of the C‐subunit in the nucleus. The Hoechst channel served to delineate the nuclear region, with the image acquired at a magnification of 63× to ensure accurate analysis.

### RNA extraction from primary fibroblast

Primary human Fibroblasts were washed with PBS before total RNA extraction using the RNeasy Plus Mini Kit (Qiagen, Germantown, MD, USA).

### RNA sequencing and analysis

The Integrity of the extracted RNA was assessed at the Genome Technology Center, Faculty of Medicine, Bar‐Ilan University, utilizing the Agilent High Sensitivity RNA Kit in combination with Tapestation 4200. A total of 500 ng of RNA was used to enrich mRNA via the NEBNext mRNA polyA Isolation Module (NEB, #E7490L, Ipswich, MA, USA). Library preparation for Illumina sequencing followed the protocol of the NEBNext Ultra II RNA kit (NEB, #E7770L). Quantification of the library was performed using the dsDNA HS Assay Kit and Qubit 2.0 (Molecular Probes Eugene, OR, USA), Life Technologies, Carlsbad, CA, USA), and qualification was done using the Agilent D1000 Tapestation Kit and Tapestation 4200. For sequencing, libraries were pooled at a concentration of 400 nm, diluted to 4 nm with NextSeq manufacturer's instructions, and loaded onto the Flow Cell at 1.6 pm with 1% PhiX library control. The sequencing process was conducted on an Illumina NextSeq 500 instrument, generating 75 cycles of single read sequencing. Sequencing data was aligned and normalized at The Nancy and Stephen Grand Israel National Center for Personalized Medicine at the Weizmann Institute of Science. Pathways and Pathological analyses were performed using the ShinyGO/Metascape web‐based tools.

### Time‐dependent BRET^2^ measurements

C‐terminal RLuc8‐tagged human RIβ‐WT and ‐L50R as well as N‐terminal GFP^2^‐tagged human Cα were used for *in vivo* BRET^2^ analysis. Time‐dependent BRET^2^ measurements were performed at 37 °C at the plate reader POLARstar Omega device (BMG LABTECH, Ortenberg, Baden‐Württemberg, Germany). Emission from the RLuc8 (at 410 nm) and GFP^2^ (at 515 nm) signals was detected and the ratio between the GFP^2^ and RLuc8 signal (BRET^2^‐ratio) was calculated. For each construct, 6 wells were measured, and the mean values of the BRET^2^‐ratio were plotted against time. In the first 4.5 min of the measurements, the signal without any stimulus was recorded. After 4.5 min of detection, different cAMP stimuli were applied to the cells to induce holoenzyme dissociation. Holoenzyme dissociation was achieved using two different stimuli that increase the intracellular cAMP concentration. Cells were first washed with HBSS buffer (Hank's Balanced Salt Solution w/o Mg^2+^/Ca^2+^, Biowest, Nuaillé, Maine‐et‐Loire, France) then stimulated either with 1 μm ISO (Sigma‐Aldrich) or with a combination of FSK (Sigma‐Aldrich) (50 μm) and the non‐specific IBMX (Sigma‐Aldrich) (100 μm), and the resulting signal was detected in the following 15.5 min. Coelenterazin 400a (DeepBlueCTM, DBC, Biotrend, Miramar Beach, FL, USA) in a final concentration of 5 μm per well was used as substrate for the Luciferase enzymatic reaction. All dilutions for the cAMP stimuli and DBC were prepared in HBSS buffer (Hank's Balanced Salt Solution w/o Mg^2+^/Ca^2+^, Biowest).

### Expression, purification of recombinant cα and his‐RIβ

N‐terminal His‐tagged human RIβ‐WT and RIβ‐L50R constructs, cloned in a pQTEV‐His_7_ vector, were used to express recombinant RIβ proteins for *in vitro* biochemical analysis, Including size‐exclusion chromatography, fluorescence polarization, holoenzyme activation, and SPR measurements. The human RIβ‐WT and L50R constructs were expressed in *E. coli* Δcya TP2000 cells [52, 53] to obtain cAMP‐free R‐subunits. To obtain high amounts of recombinant proteins, liters of cell cultures were inoculated with precultures of colonies from transfection and incubated at 37 °C under shaking at 150 rpm till an optical density of ~ 0.6 was reached. Protein expression was induced by adding 400 μm IPTG (Isopropyl β‐D‐1‐thiogalactopyranoside, Fermentas) following incubation overnight (at 18–19 °C). Cells were harvested at 9000 ***g*** for 30 min at 4 °C. Purification of the WT and L50R mutant was performed using a Ni^2+^‐NTA‐Agarose (Protino Ni^2+^‐NTA Agarose, Macherey‐Nagel™) via their N‐terminal polyhistidine Tags (His_7_‐Tag). All other chemicals were purchased from Roth unless otherwise mentioned. Harvested cell pellets were treated with lysis buffer containing 50 mm KH_2_PO_4_ (pH 8.0), 500 mm NaCl, 20 mm imidazole, 5 mm 2‐Mercaptoethanol, as well as freshly added 0.1% Triton X‐100 and protease inhibitor (cOmplete™ EDTA‐free protease inhibitor cocktail, Sigma‐Aldrich) and lysed via French Pressure Cell (Thermo IEC). After centrifugation at 45 000 ***g*** for 30 min (at 4 °C) supernatant was collected and applied to a previously equilibrated agarose resin. After 1 h of incubation at 4 °C, resins were washed by centrifuging the samples for 2 min at 1000 ***g*** (at 4 °C) with two different wash buffers containing increasing amounts of imidazole. After two wash steps with the first wash buffer containing 50 mm KH_2_PO_4_ (pH 8.0), 500 mm NaCl, 60 mm imidazole, 5 mm 2‐Mercaptoethanol, a last wash step was performed with the following buffer: 50 mm KH_2_PO_4_ (pH 8.0), 500 mm NaCl, 100 mm imidazole, 5 mm 2‐Mercaptoethanol. The elution of the R‐subunits was performed with 50 mm KH_2_PO_4_ (pH 8.0), 500 mm NaCl, 250 mm imidazole, 5 mm 2‐Mercaptoethanol. Each purification step was analyzed by an SDS‐Gel. Proteins were stored on ice at 4 °C in 20 mm MOPS (pH 7.4), 150 mm NaCl, 2 mm EGTA, 2 mm EDTA, and 5 mm 2‐Mercaptoethanol. Overexpression of the C‐subunit was performed in *E. coli* BL21(DE3) cells and purified with IP20 affinity chromatography as previously described [54, 55].

### Holoenzyme activation

Holoenzyme was performed by mixing 3 μm C‐subunits with a slight excess of N‐terminal His‐tagged R‐subunits in 200 μL for 1 h on ice at 4 °C in 20 mm MOPS, pH 7.4, 150 mm NaCl, 1 mm ATP, 10 mm MgCl_2_, 2 mm 2‐Mercaptoethanol, and 0.5 mg·mL^−1^ bovine serum albumin (BSA, Sigma‐Aldrich). Holoenzyme activation was determined by a coupled spectrophotometric kinase assay as described by [56], where the activity of the C‐subunit was measured by the phosphorylation of the synthetic peptide Kemptide (Leu‐Arg‐Arg‐Ala‐Ser‐Leu‐Gly) (GeneCust, Boynes, France). Measurements were performed with a 384‐well transparent microplate (BRAND #781680) in a plate reader (CLARIOstar Omaga; BMG LABTECH). For the activation measurements, holoenzyme was diluted in Cook Assay Mix containing 100 mm MOPS (pH 7.0), 10 mm MgCl_2_, 1 mm Phosphoenolpyruvate (Sigma‐Aldrich), 1 mm ATP, 15 U·mL^−1^ Lactate dehydrogenase (Roche #10127876001, Basel, Basel‐Stadt, Switzerland), 8.4 U·mL^−1^ Pyruvate kinase (Roche #10128163001), 5 mm 2‐Mercaptoethanol, and 0.2 mm NADH (Roth, Karlsruhe, Baden‐Württemberg, Germany). Different cAMP concentrations (Biolog Life Science, Bremen, Bremen, Germany) were used to induce holoenzyme dissociation, and the reaction was started by adding 13 μL of the peptide substrate Kemptide (Leu‐Arg‐Arg‐Ala‐Ser‐Leu‐Gly, GeneCust) in a final concentration of 251 μm to reach a total reaction volume of 100 μL per well with a final C‐subunit concentration of 20 nm. cAMP and Kemptide dilutions were prepared in 20 mm MOPS (pH 7.4), 2 mm 2‐Mercaptoethanol, and 0.5 mg·mL^−1^ BSA (Sigma‐Aldrich). To obtain the kinase activity, the extinction of NADH was measured at 340 nm for 13 min and plotted over time. For each cAMP concentration, duplicate measurements were performed. The obtained slope values (Δ340 nm·min^−1^) from the linear regression were then plotted against increasing cAMP concentrations. For calculation of the *K*_act_ and Hill slope values, a sigmoidal dose–response fit was applied. Data evaluation was performed with the graphpad prism 8.0.1 software (GraphPad Software, Inc, Boston, MA, USA).

### Surface plasmon resonance analysis

Interaction of RIβ‐WT or RIβ‐L50R with a FSS‐tagged hCα‐subunit was analyzed by surface plasmon resonance (SPR) using a Biacore T200 (Cytiva). FSS‐hCα was captured as ligand on a Biacore CM5‐Chip S‐series (Cytiva) previously coated with Strep‐Tactin®XT using the Twin‐Strep‐tag® Capture Kit of IBA Lifesciences GmbH (Göttingen, Germany) as described in the manual. N‐terminal His‐tagged RIβ‐subunits were used as analytes. All SPR measurements were performed in 10 mm HEPES (pH 7.4), 150 mm NaCl, 1 mm ATP, 10 mm MgCl_2_, and 0.05% Tween20 (Sigma‐Aldrich). One of the flow cells of the chip was used as a reference. On this reference cell no C‐subunit was captured. Capturing of 5 nm C‐subunit on the measurement cell (second flow cell) was performed for 50 s at a flow rate of 10 μL·min^−1^. After capturing, a series of R concentrations were applied at a flow rate of 30 μL·min^−1^ to both flow cells for 300 s; injection of buffer without R‐subunit (30 μL·min^−1^ flow rate) was performed to initiate dissociation (300 s). As multi‐cycle kinetics were performed, regeneration of the chip surface for each R concentration was induced by treating the chip three times with a 3 m Guanidinium chloride solution for 60 s at a flow rate of 30 μL·min^−1^. For all the SPR measurements, the functional R‐subunit concentration was used. For the determination of the functional R‐subunit concentration, a fixed concentration of 5 nm C‐subunit was titrated with different concentrations of cAMP‐free R‐subunits in the above‐mentioned spectrophotometric assay [56]. For analyzing the binding kinetics of C‐ and R‐subunit, the plotted SPR‐curves were fitted with a 1 : 1 Langmuir binding model (A + B ↔ AB) for calculation of association (*k*_a_) and dissociation (*k*_d_) rates as well as for the equilibrium dissociation constant (*K*_D_). Data evaluation was performed with the software Biacore T200 evaluation software 3.2 (Cytiva). graphpad prism 8.0.1 (GraphPad Software, Inc) was used for data visualization.

To test the cAMP‐triggered dissociation of the RIβ‐WT or RIβ‐L50R from the catalytic subunit, FSS‐hCα was captured to a Strep‐Tactin®XT coated CM5‐Chip as described above. WT‐ or mutant R‐subunit (15 nm) was injected over the immobilized FSS‐C subunit for 300 s and holoenzyme stability was monitored for an additional 200 s in running buffer. Subsequently, increasing concentrations of cAMP were injected in the dissociation phase for 150 s to induce holoenzyme dissociation. After each cycle (capture of FSS C binding of 15 nm R‐subunit, holoenzyme stability check, cAMP‐triggered holoenzyme dissociation), chip regeneration was performed as described above. Data were normalized before R‐subunit association (0%) and before cAMP‐triggered dissociation (100%). The evaluation was performed with the software biaevaluation 4.1.1., biacore t200 evaluation software 3.2 (Cytiva) and graphpad prism 8.0.1 (GraphPad Software, Inc).

### Size‐exclusion chromatography

To investigate dimer formation, size‐exclusion chromatography (SEC) was performed with recombinant N‐terminal His‐tagged RIβ‐WT or RIβ‐L50R using an ÄKTA go protein purification system with a Superose™ 6 Increase 3.2/300 (Cytiva). Proteins were injected on the column using a 50 μL injection loop at a flow rate of 0.04 mL·min^−1^ in 20 mm MOPS (pH 7.4), 150 mm NaCl, and 2 mm 2‐Mercaptoethanol. Measurements were performed at 4 °C. The determination of the Stokes' radii was performed using a calibration plot with protein standards from the Gel Filtration Calibration Kit LMW of (Cytiva) (Fig. 5C). For the determination of the column void volume (*v*_0_) Blue dextran 2000 (Cytiva) was used (Fig. 5C). The partition coefficients (*K*_av_) for each standard were first calculated, and the (− log_10_ *K*_av_)^1/2^ were then plotted against the respective Stokes' radii [57]. Fractions of the protein peaks of the L50R were collected and identified by western blot analysis. Detection was achieved by detecting the fluorescent signal at 800 nm using the LI‐COR odyssey® Fc device (LI‐COR Biosciences).

### Fluorescence polarization

The fluorescence polarization competition assay was performed by mixing increasing cAMP concentrations, diluted in a buffer containing 8‐(2‐[fluoresceinyl]aminoethylthio)‐cAMP (8‐Fluo‐cAMP) (final concentration 0.5 nm) with N‐terminal His‐tagged WT or L50R mutant proteins (final concentration 4 nm) in a 1 : 1 ratio as previously described [58]. For measurements, 384‐Well microplates (BRAND, #781622) were used, and each cAMP concentration was measured in duplicates. For the detection, a CLARIOstar Omega plate reader (BMG LABTECH) was used. All dilutions were performed in a buffer containing 20 mm MOPS (pH 7.4), 150 mm NaCl, 0.005% CHAPS, and 1 mm DTT (Dithiothreitol, Roche). Data were blank subtracted using the MARS evaluation software (BMG LABTECH) and analyzed to obtain EC_50_ values. For the calculation of the EC_50_ values, the sigmoidal dose–response fit in the graphpad prism 8.0.1 software (GraphPad Software, Inc) was applied.

## Conflicts of interest

The authors declare no conflict of interest.

## Author contributions

TB‐Z, VP, RS‐S, MA, AD, DB, AH, MOM, VL‐R were responsible for experiment implementation and data analysis. MS, VL‐R participated in data collection and statistical analysis on cellular images. TB‐Z, VP, DB, AH, VL‐R, FWH, RM, and RI contributed to experimental conceptualization, figure visualization, and draft writing. FWH, RM, DB, VL‐R, JvS, and RI provided methodology guidance. TB‐Z, VP wrote the manuscript with assistance from DB, FWH, and RI. All authors reviewed the results and approved the final version of the manuscript.

## Peer review

The peer review history for this article is available at https://www.webofscience.com/api/gateway/wos/peer‐review/10.1111/febs.70098.

## Data Availability

The data that support the findings of this study are available from the corresponding author [ronit.ilouz@biu.ac.il] upon reasonable request.
